# Dynamic 3D genome architecture of cotton fiber reveals subgenome-coordinated chromatin topology for 4-staged single-cell differentiation

**DOI:** 10.1186/s13059-022-02616-y

**Published:** 2022-02-03

**Authors:** Liuling Pei, Xianhui Huang, Zhenping Liu, Xuehan Tian, Jiaqi You, Jianying Li, David D. Fang, Keith Lindsey, Longfu Zhu, Xianlong Zhang, Maojun Wang

**Affiliations:** 1 National Key Laboratory of Crop Genetic Improvement, Hubei Hongshan Laboratory, Huazhong Agricultural University, Wuhan, 430070 Hubei China; 2 Cotton Fiber Bioscience Research Unit, USDA-ARS, Southern Regional Research Center, New Orleans, LA 70124 USA; 3 Department of Biosciences, Durham University, Durham, DH1 3LE UK

**Keywords:** 3D genome, Cotton fiber, Single-cell differentiation, Polyploid

## Abstract

**Background:**

Despite remarkable advances in our knowledge of epigenetically mediated transcriptional programming of cell differentiation in plants, little is known about chromatin topology and its functional implications in this process.

**Results:**

To interrogate its significance, we establish the dynamic three-dimensional (3D) genome architecture of the allotetraploid cotton fiber, representing a typical single cell undergoing staged development in plants. We show that the subgenome-relayed switching of the chromatin compartment from active to inactive is coupled with the silencing of developmentally repressed genes, pinpointing subgenome-coordinated contribution to fiber development. We identify 10,571 topologically associating domain-like (TAD-like) structures, of which 25.6% are specifically organized in different stages and 75.23% are subject to partition or fusion between two subgenomes. Notably, dissolution of intricate TAD-like structure cliques showing long-range interactions represents a prominent characteristic at the later developmental stage. Dynamic chromatin loops are found to mediate the rewiring of gene regulatory networks that exhibit a significant difference between the two subgenomes, implicating expression bias of homologous genes.

**Conclusions:**

This study sheds light on the spatial-temporal asymmetric chromatin structures of two subgenomes in the cotton fiber and offers a new insight into the regulatory orchestration of cell differentiation in plants.

**Supplementary Information:**

The online version contains supplementary material available at 10.1186/s13059-022-02616-y.

## Background

Plant cell differentiation requires changes in transcriptional regulatory networks of a variety of genes including those encoding transcription factors [1, 2]. Accumulating evidence shows that epigenetic reprogramming affects the expression of differentiation-related genes during plant cell differentiation [3, 4]. Epigenetic modifications, such as DNA methylation and histone modifications, change the chromatin environment in which transcription factors and other basic regulators act [3]. A genome-wide methylation study of maize leaves showed that DNA methylation changes significantly during the transition of cells from the proliferation stage to the differentiation stage, which influences the expression of genes involved in chromatin remodeling, cell cycle, and growth regulation [5]. During the development of *Arabidopsis* root apical meristematic cells, histone acetylation rises to the highest level in mitotic cells, and then decreases with cell differentiation [6]. Recently, a single-cell transcriptome and chromatin accessibility landscape analysis of rice root tips showed that the meristem cells undergo chromatin reprogramming during cell differentiation [7].

In recent years, the rapid development of three-dimensional (3D) genomics techniques, especially high-throughput chromosome conformation capture (Hi-C) [8] and chromatin interaction analysis based on paired-end tag sequencing (ChIA-PET) [9], uncovers the hierarchical principles of interphase chromatin 3D structure that is partitioned into chromosome territories (CTs), chromatin compartments, topologically associating domains (TADs), and loops [10]. Recent studies have also shown that chromatin interaction occurs between multiple TADs, which adds another layer of higher-order chromatin organization [11, 12]. Chromatin 3D structure is highly dynamic and is related to gene expression in a variety of biological processes [13–19]. In rice, analysis of 3D chromatin organization and dynamics before and after fertilization shows that the chromatin structures of eggs and sperm cells are similar to that of mesophyll cells and recombine after fertilization [15]. Comparison of the chromatin 3D structures in multiple tissues of rice, millet, and maize showed that the overall A/B compartment is stable among different tissues, while the local A/B compartment has tissue-specific dynamics related to differential gene expression [16]. A study in maize showed that some TAD boundaries are tissue-specific in ear and tassel, and the expression of a few genes at these tissue-specific boundaries varies [18]. In *Arabidopsis* and rice, heat stress causes a global rearrangement of the 3D genome, including A/B compartment switching, an increase in the size of TADs and loss of short-range interactions [17, 19].

Cotton fiber is a single-cell structure derived from the epidermis of the ovule and serves as a model system for studying cell differentiation in plants [20]. Cotton fiber development undergoes staged differentiation that can be divided into four overlapping stages: initiation, elongation, secondary cell wall synthesis, and maturation [21]. During these four stages, the morphological and structural changes of fiber cells are accompanied by important physiological and biochemical processes, involving a large number of genes [21]. Transcription factors play an important role in the initiation of cotton fiber cell differentiation [22]. Cell wall structure, cytoskeleton, and lipid metabolism-related genes are closely related to fiber cell elongation [23–27]. The secondary cell wall synthesis stage mainly involves cellulose synthesis and deposition, and genes such as *sucrose synthase* and *cellulose synthase* (*CesA*) play a key role in this stage [28, 29].

Epigenetic modifications are thought to contribute to cotton fiber development. DNA methylation dynamics, especially for the increase of CHH (H=A, T or C) methylation, regulates differential homoeologous gene expression during allotetraploid cotton fiber development [30, 31]. The downregulated expression of the H3K9 deacetylation gene *GhHDA5* leads to H3K9 hyperacetylation in the promoter regions of a number of genes, which inhibits fiber cell differentiation and lint production [32]. Our previous studies illustrated the evolutionary reorganization of higher-order chromatin structure in cotton leaves after polyploidization and differential amplification of transposable elements (TE) [33, 34], but little is known about 3D chromatin organization in fiber and its functional implications in staged cell differentiation.

Here we present the dynamic architecture of 3D genome organization during allotetraploid cotton fiber development. We find differential patterns of A/B compartment switching and TAD-like structure organization between the At (“t” represents “tetraploid”) and Dt subgenomes. We also found that dynamic chromatin loop-mediated divergent regulatory networks between the two subgenomes that indicate homoeologous expression bias. The resolved spatiotemporal dynamic chromatin structures provide new insights into subgenome-coordinated contribution to allotetraploid fiber development, and shed light on chromatin topology-mediated transcriptional regulation of cell differentiation in plants.

## Results

### Subgenome-coordinated contribution to allotetraploid fiber development

**Fig. 1:**
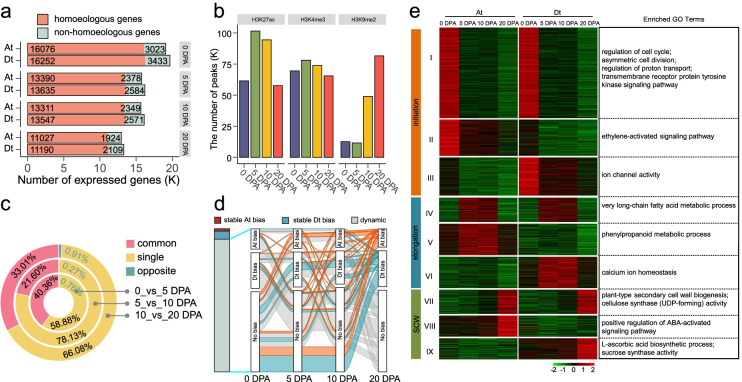
Coordinated contribution of At and Dt subgenomes to *G. barbadense* fiber development. **a** The bar plot shows that the number of expressed genes in the At and Dt subgenomes at 0 day post anthesis (DPA), 5 DPA, 10 DPA, and 20 DPA. The expressed genes contain homoeologous genes (orange) and non-homoeologous genes (cyan). **b** The bar plot shows the number of peaks for different chromatin modifications at four stages. **c** The circular diagram shows the proportion of differential expression patterns of homoeologous gene pairs in the comparisons of adjacent stages. **d** Dynamic number of homoeologous genes with expression bias during fiber development. Conserved At bias means that the expression levels of homoeologous genes in the At subgenome were significantly higher (FC ≥ 2 and FDR ≤ 0.05) than those in the Dt subgenome at the four stages. Conserved Dt bias shows the opposite meaning to conserved At bias. Dynamic category represents dynamic changes of the expression bias towards the At or Dt subgenome. Cyan line indicates genes showing Dt bias expression, orange line indicates genes showing At bias expression and gray line indicates genes with no bias expression at 20 DPA. **e** Heatmap shows the clustering of homoeologous expression patterns into nine groups. The representative GO terms are shown for each group. High expression is shown in red and low expression is shown in green

We collected cotton ovule at 0 day post anthesis (DPA) and fiber samples at 5 DPA, 10 DPA, and 20 DPA from *G. barbadense* 3-79, representing fiber initiation, elongation (early elongation and fast elongation) and secondary cell wall synthesis stages respectively. These samples were used for RNA-seq and ChIP-seq analysis (H3K27ac, H3K4me3, and H3K9me2) to explore dynamic gene expression and chromatin modifications (Additional file 1: Table S1). It was found that during fiber development, the total number of expressed genes decreased and the number of expressed genes in the Dt subgenome was slightly greater than in the At subgenome (Fig. 1a; Additional file 2: Figs. S1a, b). Active histone modifications (H3K27ac and H3K4me3, two representative modifications involved in gene transcription) had the largest numbers of peaks at 5 DPA and decreased during development; repressed modification (H3K9me2, a representative modification deposited in heterochromatin) had the lowest number of peaks at 5 DPA and gradually increased (Fig. 1b; Additional file 1: Table S1; Additional file 2: Figs. S1c-e). In total, we identified 10,155 (0 vs 5 DPA), 2162 (5 vs 10 DPA), and 7188 (10 vs 20 DPA) differentially expressed genes in three comparisons during fiber development (Additional file 3: Table S2). These data suggest that fewer genes were involved in fiber development as fiber elongation progresses, accompanied by a decrease in active chromatin modifications.

At the homoeologous gene level, the expression pattern changes were classified into three categories: single change (only one of the homoeologous gene pairs in the At or Dt subgenome exhibited differential expression in fiber development), common change (both homoeologous genes were up- or downregulated simultaneously), and opposite change (the expression changes of homoeologous genes exhibited opposite patterns). It is found that the homoeologous genes categorized as single change were most common; the homoeologous genes categorized as opposite change were the least (Fig. 1c). Interestingly, 7970 homoeologous genes showed dynamic changes in terms of expression bias between the At and the Dt subgenomes (Fig. 1d; Additional file 4: Table S3). The expression patterns of homoeologous genes were clustered into 14 groups (Additional file 2: Fig. S2; Additional file 5: Table S4), among which 12,470 were specifically expressed in some stages of fiber development (Fig. 1e). The genes in groups I–III were specifically expressed at the initiation stage; the genes in groups IV–VI were specifically expressed at the elongation stage; the genes in groups VII–IX were specifically expressed at the secondary cell wall synthesis stage. Gene Ontology (GO) enrichment analysis showed that a few different biological processes related to fiber development were enriched in these subgenome-biased groups. These data suggest that, to a large degree, the At and Dt subgenomes had functionally coordinated contributions to allotetraploid fiber development (Fig. 1e).

### Dynamic 3D genome architecture of fiber development

**Fig. 2:**
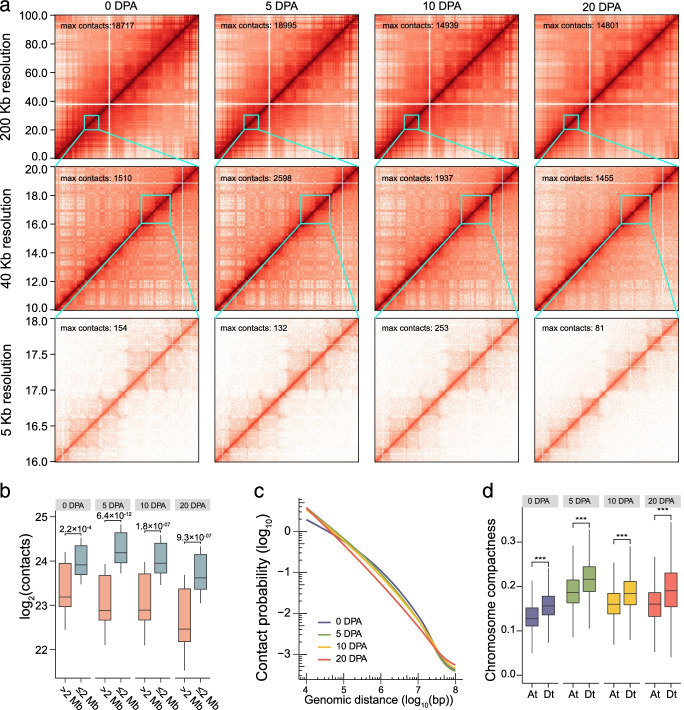
The high-resolution landscapes of chromatin interaction during fiber development. **a** Multi-resolution Hi-C interaction heatmaps at different stages of fiber development. Hi-C matrices were constructed at resolutions of 200 kb (0–100 Mb in chromosome A02), 40 kb (10–20 Mb in chromosome A02), and 5 kb (16–18 Mb in chromosome A02). The Hi-C data were generated at 0, 5, 10, and 20 DPA. **b** The total number of Hi-C contacts for short-range (≤ 2 Mb) and long-range (> 2 Mb) chromatin interactions. **c** The chromatin contact probabilities relative to the genomic distance from 1 kb to 100 Mb. **d** Box plots showing the comparison of chromatin compactness between the At and Dt subgenomes. For both **b** and **d**, two-sided Wilcoxon signed-rank test was used (***P < 2.2 × 10^−16^)

To investigate the chromatin topology and its regulation of fiber development, we performed in situ Hi-C to reveal the dynamics of chromatin conformation using the same samples described above. Considering the high reproducibility of biological replicates for each Hi-C experiment, we combined both biological replicates for downstream analyses (Additional file 2: Fig. S3a). In total, we obtained 689.63, 663.90, 694.78, and 715.70 million uniquely aligned contact reads for each respective developmental stage (Additional file 6: Table S5). The data allowed us to construct chromatin interaction matrices reaching a 5-kb resolution, evaluated using a previous method (Additional file 2: Fig. S3b) [35]. At different resolutions, different higher-order chromatin structures appeared (Fig. 2a; Additional file 2: Fig. S4).

During fiber development, the number of intrachromosomal interaction contacts decreased and the number of interchromosomal interactions increased (Additional file 2: Fig. S5). At the chromosomal level, more interactions occurred in a short distance (≤ 2 Mb) (Fig. 2b). We calculated chromatin contact probability to study the change in chromatin interaction strength with increased linear distance. A general negative correlation was obtained (Fig. 2c). We found that the extent of chromatin compactness increased in fiber samples relative to cotton ovule (0 DPA) (Fig. 2d). Meanwhile, we analyzed the interaction spanning distance, contact probability, and chromatin compactness in gene-poor and gene-rich regions, respectively. The result is consistent with the findings above (Additional file 2: Fig. S6). The comparison between the At and Dt subgenomes revealed that the Dt subgenome was more highly compacted than the At subgenome, and this difference was pronounced in gene-poor regions (Additional file 2: Fig. S6c). The established dynamic 3D genome architecture could serve as a resource for uncovering the higher-order chromatin structures and transcriptional regulation in fiber development.

### Switching of A/B compartment status in the At and Dt subgenomes

**Fig. 3:**
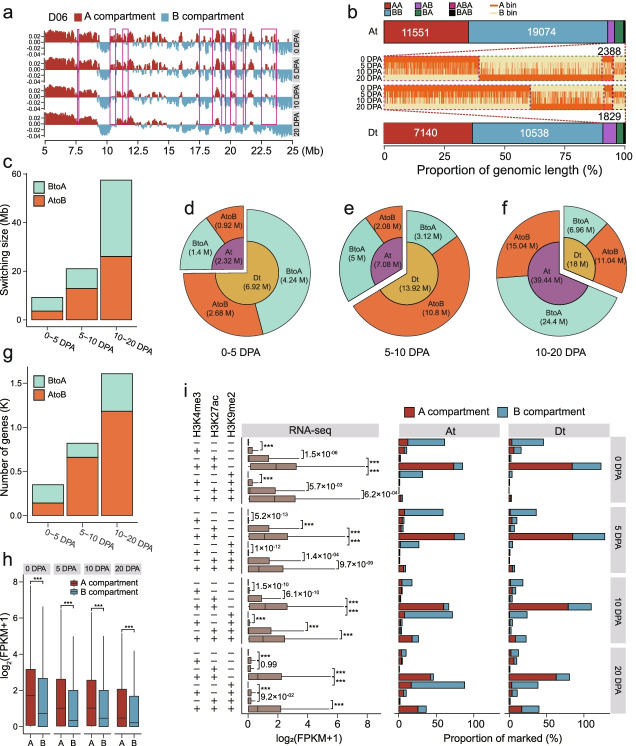
Switching of A/B compartment status in the At and Dt subgenomes. **a** One representative genomic region showing the dynamic switching of A/B compartment status in chromosome D06. Positive values represent A compartment and negative values represent B compartment. Some regions exhibiting A/B compartment switching are indicated in pink boxes. **b** Global dynamic switching of chromatin compartment status. Chromosomal bins with stable chromatin status are shown in red (A compartment) or turquoise (B compartment). Heatmaps show bins with status switching between A compartment and B compartment. “AB” shows switching from A to B, “BA” shows switching from B to A (include BAAA, BABA, BBAA, BBBA), “ABA” shows two switching from A to B and then from B to A (include ABBA, ABAA, AABA), “BAB” shows two switching from B to A and then from A to B. **c** The size of the A/B compartment switching regions between adjacent stages. **d–f** The size of the A/B compartment switching regions at the subgenome level between adjacent stages. **g** The number of genes contained in the A/B compartment switching regions. **h** Comparison of the expression level of genes contained in the A/B compartment regions. **i** Box plot showing the expression level of genes located in regions marked by different chromatin modifications. Bar plot showing the proportion of A/B compartment regions marked by different modifications. ‘-’not marked, ‘+’ marked. For both **h** and **i**, two-sided Wilcoxon signed-rank test was used (***P < 2.2 × 10^−16^)

We categorized the chromatin at each developmental stage using 40-kb Hi-C matrix into A and B compartments representing active and inactive chromatin regions respectively. It is found that the total genomic lengths of the A and B compartments are similar (Additional file 2: Fig. S7a). We investigated the dynamic switching of A/B compartment status at four developmental stages (Fig. 3a; Additional file 2: Fig. S7b). At the global level, the status of most chromatin compartments, including 92.77% of At subgenome and 90.62% of Dt subgenome, was stable (Fig. 3b). The switching of A/B compartments in the At subgenome was compared with that in the Dt subgenome. More A compartment switched into B compartment in the Dt subgenome from 0 to 20 DPA (marked by purple dashed box), the opposite to what was observed in the At subgenome (marked by green dashed box) (Fig. 3b). For the dynamic switching regions, we observed that more regions switched into A compartment in the comparisons of 0–5 DPA and 10–20 DPA, while the ratio of B compartment became larger in the comparison of 5–10 DPA (Fig. 3c).

The comparison of genomic sizes with A/B compartment switching showed that the Dt subgenome had more regions (4.24 Mb) switching into A compartment (BtoA) than the At subgenome (1.4 Mb) from 0 to 5 DPA, while the At subgenome had more B to A switching (24.4 Mb) than the Dt subgenome (6.96 Mb) between 10 and 20 DPA (Fig. 3d–f). Overall, the Dt subgenome had more A compartment regions switched to B compartment status, which was reversed in the At subgenome (Fig. 3b; Additional file 2: Fig. S7c). These data suggest that many Dt-subgenomic regions at the early developmental stage were active, while more At subgenomic regions became active at the later developmental stage. Even though the total genomic length of B to A switching in the comparison of 10–20 DPA was larger than that of A to B switching, more genes exhibited A to B switching, indicating that many regions with B to A switching contained fewer genes (Fig. 3g; Additional file 2: Figs. S8-11). In total, 861 and 1796 genes were located in regions with A/B compartment switching in the At and Dt subgenomes, respectively (Additional file 2: Fig. S11a; Additional file 7: Table S6). Genes in regions with compartment switching were enriched in some different biological processes (Additional file 8: Table S7). These results further confirm that the At and Dt subgenomes have coordinated roles in the whole developmental process.

At the expression level, genes located in the A compartment had higher expression levels than genes in the B compartment (Fig. 3h), and the A compartment regions had a higher gene density than the B compartment (Additional file 2: Fig. S11b). We further found that the genes located in regions exhibiting A to B switching had a probability of downregulated expression and the genes in regions of B to A tended to exhibit upregulated expression (Additional file 2: Fig. S11c). This result is similar to previous studies [36, 37], suggesting that large-scale 3D structural changes correlate with gene expression. Notably, in combining ChIP-Seq with RNA-seq data, we observed that genes contained in H3K4me3- or H3K27ac-marked regions exhibited generally higher expression levels than genes located in H3K9me2-marked regions (Fig. 3i). Active regions (marked by H3k4me3 or H3K27ac) tended to be included in the A compartment and inactive regions (marked by H3K9me2) tended to be included in the B compartment (Fig. 3i). As expected, the proportion of active regions decreased at the latter developmental stage, consistent with the decreased number of genes expressed (Fig. 3i).

### Dynamic organization of TAD-like structure in fiber development

**Fig. 4:**
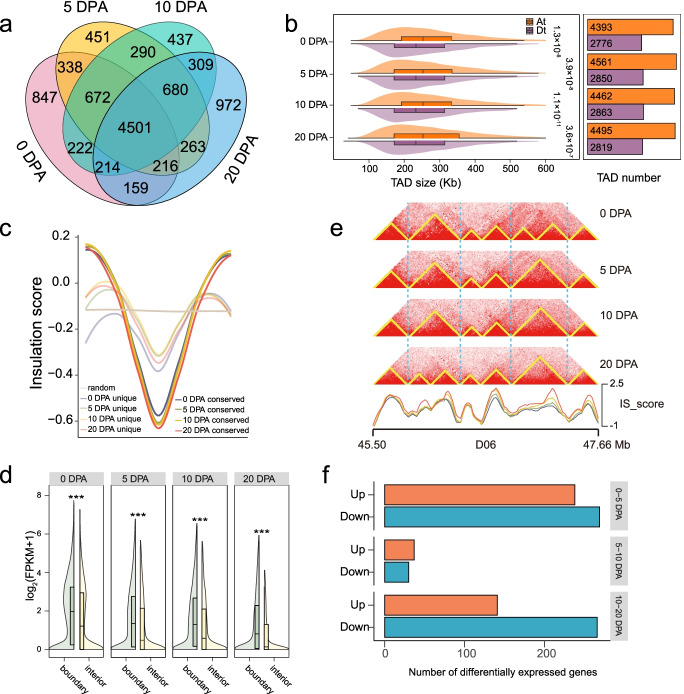
Global dynamic TAD-like structure organization during fiber development. **a** Venn diagram showing the number of conserved or specific TAD-like structure at four stages: 0 DPA (red), 5 DPA (yellow), 10 DPA (cyan), 20 DPA (blue). **b** Box plot showing the distribution of structure size in the At and Dt subgenomes. Bar plot showing the structure number. **c** The average insulation scores at conserved and unique boundaries. The scores in randomly distributed regions were shown as a control. **d** The expression level of genes located in boundary or interior. **e** Heatmaps showing representative dynamic TAD-like structure organization in fiber development, with insulation scores shown below. **f** Bar plot showing the number of expressed genes in dynamic boundaries. For both **b** and **e**, two-sided Wilcoxon signed-rank test was used (***P < 2.2 × 10^−16^)

The reorganization of TADs will lead to the change of chromatin interaction around TAD boundaries, which may affect the expression of genes [38, 39]. Using the 20-kb resolution matrices, we identified a total of 10,571 TAD-like structures including 7169, 7411, 7325, and 7314 at 0, 5, 10, and 20 DPA respectively (Additional file 9: Table S8). The comparison at different stages showed that 4501 TAD-like structures (42.58%) were thoroughly conserved at four stages, 3363 (31.82%) are relatively conserved (conserved at two or three stages), and 2707 (25.6%) were completely stage-specific, including 847 at 0 DPA, 451 at 5 DPA, 437 at 10 DPA, and 972 at 20 DPA (Fig. 4a). The percentages of conserved TAD-like structures in the two subgenomes were 73.84% for the At and 75.29% for the Dt (Additional file 2: Fig. S12). At each stage, we found that both the number and size of TAD-like structures in the At subgenome were generally larger than those in the Dt subgenome (Fig. 4b; Additional file 2: Fig. S13), possibly associated with the larger genome size of the At. Insulation score was used as a measure to separate different TAD-like structures (Fig. 4c). We found that conserved boundaries of TAD-like structures had more robust chromatin insulation than dynamic boundaries during fiber development, consistent with previous studies [38].

We next interrogated the effect of TAD-like structure organization on gene transcription. It was found that many genes are preferentially located in boundaries, and genes in boundaries exhibit higher expression levels than those in interiors at each stage (Fig. 4d; Additional file 2: Fig. S14). During fiber development, some TAD-like structures remain stable and some change dynamically (Fig. 4e; Additional file 2: Fig. S15). We explored the relationship between structure reorganization, i.e., boundary changes, and gene expression change during fiber development. The result indicates that changes in boundaries at 0–5 DPA and 10–20 DPA are associated with more altered gene expression than at 5–10 DPA (Fig. 4f). Specifically, the differential expression of 507 (0 vs 5 DPA), 67 (5 vs 10 DPA), and 407 (10 vs 20 DPA) genes were observed with TAD-like structure reorganization (Additional file 10: Table S9). These differentially expressed genes were enriched in a few biological processes, such as carbohydrate metabolic process and sucrose metabolic process (Additional file 2: Fig. S16). These analyses document the details of TAD-like structure reorganization during fiber development, pointing to the study of transcriptional regulation of genes around boundaries.

### Differential TAD-like structure organization between the At and Dt subgenomes

**Fig. 5:**
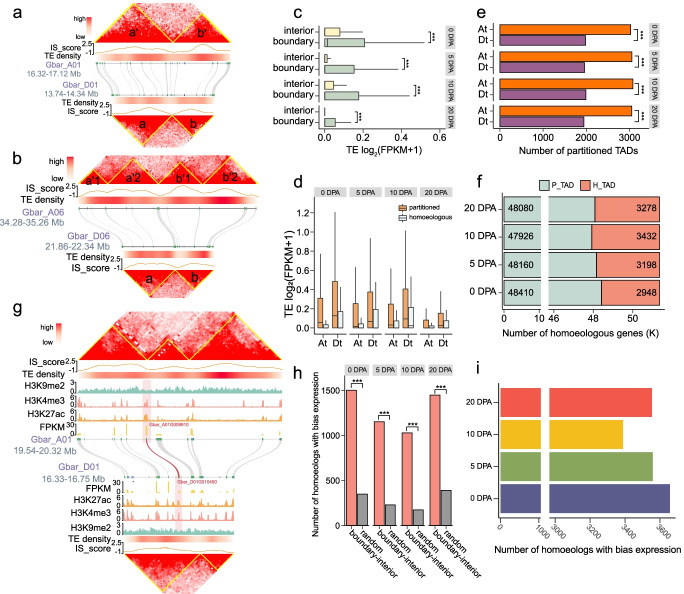
Differential TAD-like structure organization between the At and Dt subgenomes. **a,b** The homoeologous TAD-like structure between the At and Dt subgenomes contain homoeologous genes. Horizontal heatmaps show the transposable elements (TE) density. Homoeologous genes (green) are connected using gray lines. Non-homoeologous genes (blue) are not connected. **c** The expression level of TE located in boundary or interior. Two-sided Wilcoxon signed-rank test, ***P < 2.2 × 10^−16^. **d** Comparison of the expression level of TE contained in boundaries of homoeologous or partitioned TAD-like structure between two subgenomes. **e** The number of partitioned TAD-like structure in the At and Dt subgenomes. Pearson’s chi-squared test, ***P < 0.001. **f** The number of homoeologous genes contained in homoeologous TAD-like structure (H_TAD) and partitioned TAD-like structure (P_TAD). **g** Biased expression of homoeologous genes with topological change from interior in the Dt subgenome to boundary in the At subgenome. **h** The number of homoeologous genes showing expression bias that are located at different spatial positions relative to boundary and interior between two subgenomes. The number is compared with randomly distributed homoeologous genes showing expression bias. Pearson’s chi-squared test, ***P < 0.001. **i** Bar plot showing the number of homoeologous genes located in partitioned TAD-like structure with biased expression

Given the different sizes between the At and Dt subgenomes (At: 1.35 Gb, Dt: 787 Mb) contributed by the differential activity of transposable elements (TE) [40–42], we next investigated the extent of differential TAD-like structure organization between the two subgenomes. We defined subgenomic TAD-like structure as homoeologous structure and partitioned structure based on the consistency of the homoeologous genes contained in two subgenomes (Fig. 5a, b). There were 660, 780, 738, and 774 homoeologous TAD-like structure at 0, 5, 10, and 20 DPA, respectively (Additional file 11: Table S10). A comparison of the expression levels of TE between boundary and interior showed that boundary-located TE exhibited higher expression levels; nevertheless, TE were mainly concentrated in the interior regions, consistent with the observation of genome-wide gene expression and distribution (Figs. 4d and 5c; Additional file 2: Fig. S14). Notably, the expression of TE in boundaries of partitioned TAD-like structure was higher than that of homoeologous structure between the two subgenomes (Fig. 5d). This suggests that TE insertion/elimination was associated with the turnover of subgenome-specific boundaries that led to structure partition/fusion between the two subgenomes.

A comparison between the At and Dt subgenomes showed that the At subgenome had more partitioned structure than the Dt subgenome (Fig. 5e; Additional file 12: Table S11). This may be because the At subgenome had more TE amplification, affecting the local spatial structure to a greater extent and caused the structure to partition (Additional file 2: Fig. S17a). In terms of the number of homoeologous genes contained in homoeologous structure and partitioned structure, we found that homoeologous structure at 10 DPA contained the most homoeologous genes at four stages (Fig. 5f).

We next investigated the effect of differential TAD-like structure organization on the expression of homoeologous genes (Fig. 5a, b). It is found that homoeologous genes with expression bias are preferentially located in regions with changes in spatial location of structure organization (Fig. 5h). In total, 3657, 3559, 3389, and 3554 homoeologous gene pairs that had biased expression were contained in partitioned structure at 0, 5, 10, and 20 DPA, respectively (Fig. 5i; Additional file 2: Figs. S17b, c). As a proof of concept, a pair of homoeologous genes (Gbar_A01G009910 and Gbar_D01G010450) was shown in the corresponding boundaries of the At and Dt subgenomes. The At subgenomic gene Gbar_A01G009910 showed a higher expression level than the Dt subgenomic gene Gbar_D01G010450 (At-biased expression) (Fig. 5g). Taken together, these data suggest that differential TE activity is linked to altered 3D structure of chromatin in homoeologous chromosomes and has led to the change of spatial location of homoeologous genes, which is coupled with expression bias.

### Four-dimensional genome conformation represented by TAD-like structure cliques

**Fig. 6:**
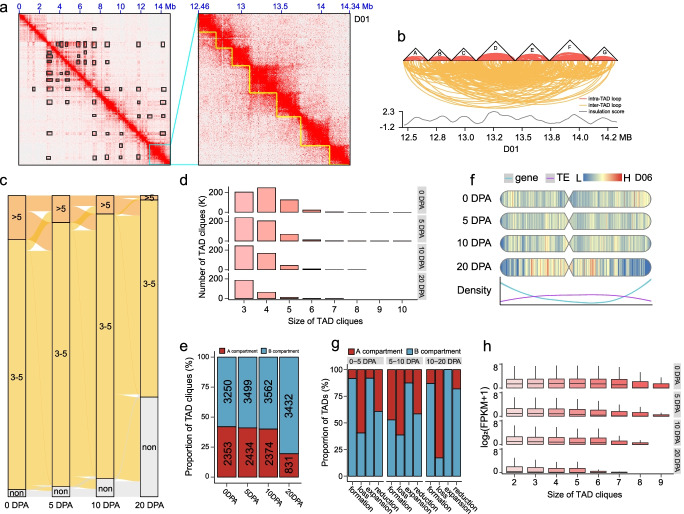
The dynamic organization of TAD-like structure cliques during fiber development. **a** Heatmap showing chromatin interaction within the range of 0–14.34 Mb (with an zoom-in region of 12.46–14.34 Mb) in chromosome D01. Black boxes represent hot regions showing TAD-TAD interactions. **b** Pattern plot showing interactions in 12.46–14.34 Mb in chromosome D01. **c** Sankey plot showing reorganization of cliques at four stages during fiber development. **d** Bar plot showing the number of cliques with different sizes. **e** Proportion and numbers of cliques in A and B compartments. **f** Heatmap showing the intensity of TAD-TAD interaction. **g** Proportion of TAD-like structure involved in clique formation, loss, expansion, or reduction in A and B compartments. **h** Box plot showing the expression level of genes contained in cliques at different scales

TAD cliques constitute higher-order chromatin assemblies that were defined for clusters of interacting TADs with numbers of greater than three (*k* ≥ 3), representing a larger scale of chromatin organization implicating transcription regulation [43]. Based on the observation of widespread chromatin interactions over the distance of TAD-like structure in our data, we used the inter-TAD-like structure interactions, which were standardized to eliminate the impact caused by the difference in size, as a standard to identify TAD-like structure cliques (Fig. 6a, b; Additional file 2: Fig. S18a). In total, 2684–6092 cliques containing 4704–7204 TAD-like structures were identified. These cliques occupied 62.59–97.12% of the genome (Additional file 2: Fig. S18b; Additional file 13: Table S12). During fiber development, the number of cliques decreased and the number of non-cliques increased. Meanwhile, the number of intricate cliques with a large interaction degree (> 5) decreased, suggesting that many cliques were subject to dissolution during fiber development (Fig. 6c, d).

To explore the functional role of TAD-like structure cliques, we classified them into active cliques located in the A compartment and inactive cliques located in the B compartment. We found that more cliques at each stage were located in the B compartment, and the proportion of cliques in the B compartment increased during fiber development. In particular, the proportion of inactive cliques increased from 10 to 20 DPA (Fig. 6e, f; Additional file 2: Fig. S18c, S19). These data indicate that many active cliques might be subject to disassembly at the later developmental stage. A further detailed analysis of dynamic cliques showed that loss of cliques (change from cliques to non-cliques) mainly occurred in the A compartment; formation of cliques (change from non-cliques to cliques) and expansion of cliques (change from 3 ≤ *k* ≤ 5 to *k* > 5) mostly occurred in the B compartment, and reduction of cliques (change from *k* > 5 to 3 ≤ *k* ≤ 5) mainly occurred in the B compartment, with similar changes between the comparisons of 0–5 DPA and 5–10 DPA and a large change in 10–20 DPA (Fig. 6g). As expected, analysis of the relationship between the size of cliques and the expression level of genes showed a negative correlation at each developmental stage (Fig. 6h). Collectively, these results suggest that TAD-like structure interactions represented by cliques preferentially occur in the B compartment and are coupled with the silencing of development-related genes in cotton fiber.

### Regulatory rewiring mediated by dynamic chromatin loops

**Fig. 7:**
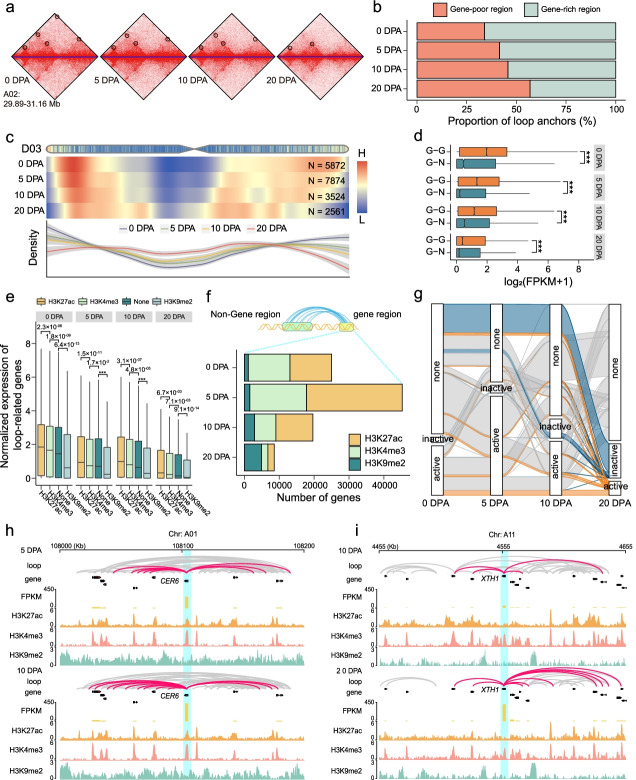
Chromatin loops implicate transcriptional regulation during fiber development. **a** Heatmap plot showing the dynamic change of chromatin loops during fiber development. Loops are marked by black circles. **b** Bar plot depicting the normalized proportion of loop anchors in gene-poor (gene number ≤ 20 in 500 kb) or gene-rich (gene number > 20 in 500 kb) regions. **c** Heatmap and line plot showing the density of loops in chromosome D03 during fiber development. N shows the number of loops. **d** Box plot showing the expression level of genes that are connected by G-G loops or G-N loops. **e** Box plot showing the expression level of genes connected with non-gene regions with different histone modifications. **f** Number of genes connected with non-gene regions with different histone modifications. **g** Dynamics of histone modifications in non-gene regions linked with genes. **h** Chromatin contacts, expression levels, and epigenetic states around 100 kb of the *XTH1* gene at 0 and 5 DPA. The red lines represent loops related to *XTH1*. **i** Chromatin contacts, expression levels, and epigenetic states around 100 kb of the *CER6* gene at 10 and 20 DPA. The red lines represent loops related to *CER6*. For both **d** and **e**, two-sided Wilcoxon signed-rank test was used (***P < 2.2 × 10^−16^)

The formation of chromatin loops allows two relatively distant anchor regions in linear chromatin to become close in the 3D conformation [44, 45]. This can facilitate the interaction of distal regulatory elements, such as promoter-enhancer or promoter-silencer regions, to efficiently regulate the expression of target genes [46, 47]. Using the 5-kb resolution matrices, we identified 289,773, 374,418, 196,828, 151,180 loops at 0, 5, 10, and 20 DPA, respectively (Additional file 2: Figs. S20a, S21, S22), in which 178,509 (23.36%) were conserved at different stages, suggesting that chromatin loops are highly dynamic. The number of loops decreased in both At and Dt subgenomes during fiber elongation (Fig. 7a; Additional file 2: Fig. S20b). The loop size and loop number of the At subgenome were larger than those in the Dt subgenome (Additional file 2: Fig. S20b). The proportion of loop anchors in gene-rich regions (gene number > 20 in 500 kb) decreased and the loop frequency in gene-poor regions (gene number ≤ 20 in 500 kb) increased (Fig. 7b), consistent with the distribution of loops in the A/B compartment (Additional file 2: Fig. S20c). At the chromosomal level, the loop density at both chromosome ends decreased while the frequency of loop interaction around centromeres increased (Fig. 7c; Additional file 2: Fig. S23). These results indicate that many chromatin interactions in active genomic regions are lost during fiber development.

Gene-centered chromatin loops were investigated to examine their potential regulatory role in transcription. We observed a positive relationship between the number of loops and the expression levels of loop-associated genes at 0 and 5 DPA, and a negative relationship at 10 and 20 DPA (Additional file 2: Fig. S24). To explore the possible reason for this occurrence, we divided gene-associated loops into two types: G-G loop (gene-gene interaction loop) and G-N loop (gene-non-gene loop) (Additional file 2: Fig. S25). The proportion of G-G loops decreased and G-N loops increased during fiber development, with 74.72% and 76.55% identified at each respective developmental stage (Additional file 2: Fig. S26). We then compared the expression levels of genes associated with G-G loops and G-N loops. It was found that the genes linked by G-G loops exhibit higher expression levels than those linked by G-N loops (Fig. 7d). Meanwhile, a positive correlation was observed between the number of G-G loops and the expression levels of genes, while a negative correlation was found for G-N loops (Additional file 2: Figs. S27-29).

To explore why G-N loops were correlated with low expression at later developmental stages, we investigated the chromatin modifications in these loop-associated non-gene regions. We found that genes with loops linked to active modifications (H3K27ac and H3K4me3) marked non-gene regions exhibit higher expression levels than those with inactive modification (H3K9me2) (Fig. 7e). The number of genes with loops linked to non-gene regions marked by active modifications decreased from 5 to 20 DPA, while the number associated with inactive modification marked non-gene regions increased (Fig. 7f; Additional file 2: Fig. S30). Meanwhile, many non-gene regions lost active modifications but gained inactive modification during fiber development (Fig. 7g). The deposition of inactive modifications and removal of active modifications might explain why more G-N loops at the later developmental stage were linked to lower expression levels of those loop-involved genes.

As a proof of principle, dynamic loops involving a few genes responsible for fiber development are shown in Fig. 7h, i. *CER6* (*3-ketoacyl-CoA synthase 6*) encodes a 3-ketoacyl-CoA synthase that can promote cotton fiber elongation [23]. The result showed that this gene gained a few G-G loops from 5 to 10 DPA, accompanied by upregulated expression (Fig. 7h). *XTH1* (*xyloglucan endotransglycosylases/hydrolase 1*) encodes a xyloglucan hydrolase related to the expansion of secondary cell wall [48]. It is found that new G-G loops related to *XTH1* were formed and some G-N loops were lost from 10 to 20 DPA (Fig. 7i). Some other examples are shown for *F3H* (*flavanone 3-hydroxylase*) and *F3'H* (*flavonoid 3'-hydroxylase*) [49] (Additional file 2: Figs. S31, S32). These data together reveal the dynamic rewiring of chromatin loops and inform future study to explore their putative regulatory role in gene expression.

### Divergent regulatory networks between the At and Dt subgenomes

**Fig. 8:**
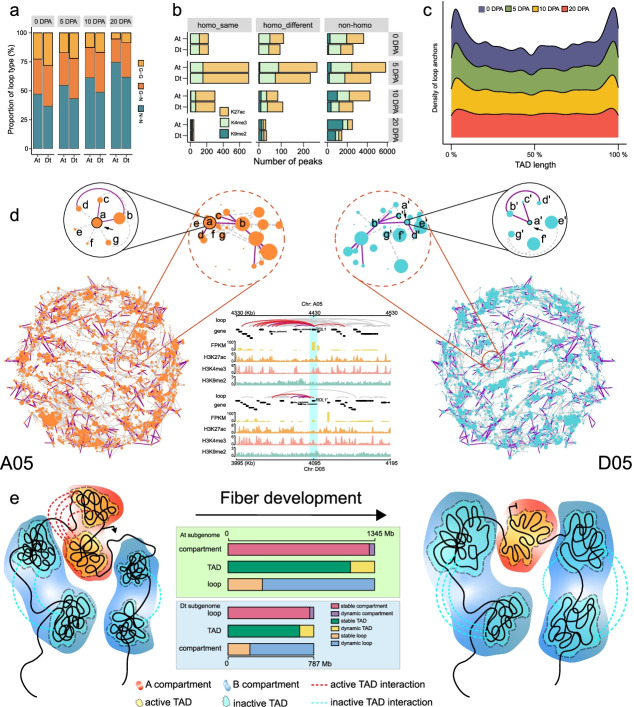
Differential regulatory networks mediated by chromatin loops between the At and Dt subgenomes. **a** Bar plot showing the proportion of different types of loops. G-G means that both loop anchors contain genes. G-N means that one of loop anchors contains genes. N-N means that both loop anchors do not contain genes. **b** Bar plot showing the number of different histone modifications in non-gene regions connected by three types of loops between the At and Dt subgenomes. **c** The density distribution of HG-HG loop anchors at the TAD-like structure level during fiber development. **d** The HG-HG loop-mediated interaction networks for homoeologous genes in chromosomes A05 and D05. The Dt-subgenomic network is mirror-symmetric to the At subgenome network. The position of each node in the At subgenomic network is consistent with that in the Dt-subgenome; the node size and network edges may be different. Each node represents a homoeologous gene and each line represents a loop (homoeologous loops in purple, non-homoeologous loops in gray) between two homoeologous genes. The node size is scaled to the number of loops associated with each gene. The purple line refers to homoeologous loops. The zoom-in plot shows the loop interactions of homoeologous gene pairs within 100 kb around At subgenomic *RDL1* (a) and Dt-subgenomic *RDL1'* (a'). The red lines represent loops associated with *RDL* and *RDL'*. **e** A proposed model showing the dynamic 3D chromatin organization during fiber development. Dynamic changes include the decreased proportion of A compartment, interactions between active TAD-like structure and chromatin loops. The bar plot shows dynamics at different scales: proportions of compartment and TAD-like structure relative to genome length, and loop number

We have shown that differences in TE amplification lead to differential TAD-like structure organization between the two subgenomes, linked to homoeologous gene expression bias (Fig. 5g, h). To further illustrate how finer chromatin 3D structures are associated with homoeologous gene expression, we show that the proportion of chromatin loop-related genes decreases, with a higher proportion in the Dt subgenome than in the At subgenome (Fig. 8a). We classified chromatin loops into two types based on the linked homoeologous genes that were defined as homoeologous loops: homoeologous gene interaction loops (HG-HG), and homoeologous gene and non-gene region interaction loops (HG-HN). HG-HG represents where both loop anchors correspond to homoeologous genes. It is found that the numbers of both HG-HG and HG-HN loops decreased from 5 to 20 DPA (Additional file 2: Figs. S33a, b), and 992 homoeologous genes were linked by stable homoeologous loops at all developmental stages (Additional file 2: Fig. S33c). HG-HN loops were further categorized into three types based on the sequence homology of non-gene regions between two subgenomes and the consistency of histone modifications: homo_same loops representing homoeologous non-gene regions with the same histone modifications, homo_different loops with different histone modifications, and non-homo loops that connected non-homoeologous non-gene sequences. We found that homoeologous genes in the At subgenome were able to interact with more non-gene regions compared with those in the Dt subgenome, and the number of interactions with H3K9me2-modified genes or non-gene regions increased in both subgenomes during fiber development (Fig. 8b).

To investigate the possible role of homoeologous loops in transcriptional regulation, we analyzed the distribution density of homoeologous loops at the TAD-like structure level. The result showed that homoeologous genes located in boundaries were more likely to be linked by loops and the loop density around boundaries was reduced during fiber development (Fig. 8c). Based on the relationship between chromatin loops (including G-G and G-N loops) and gene expression level (Fig. 7d; Additional file 2: Fig. S28), these results suggest that the expression differences between homoeologous genes may be related to differences between homoeologous loops associated with their spatial distribution in TAD-like structure. In total, there were 1466, 1431, 471, and 148 homoeologous genes showing expression bias connected with homoeologous loops (Additional file 2: Fig. S33d).

We then constructed chromatin interaction networks using HG-HG loops connecting two homoeologous genes (57,105, 54,999, 20,201, and 6714 loops at 0, 5, 10, and 20 DPA respectively), in which *RDL1* (*Response to Dehydration 22 Like 1*) related to cell wall loosening was highlighted as an example (Fig. 8d) [50]. The At subgenomic *RDL1* participated in more chromatin loops and exhibited a higher expression level than its Dt-subgenomic homoeolog (*RDL1'*). Overall, 73.26%, 72.61%, 80.93%, and 91.25% of interactions in the Dt-subgenome network were distinguished from those in the At subgenome at 0, 5, 10, and 20 DPA, respectively (Additional file 2: Fig. S34). Due to the decrease of HG-HG loops, the interaction networks became simplified at the later developmental stage.

In summary, our data shows that the spatial chromatin structure of both subgenomes is subject to reorganization, represented by the decrease of active TAD-like structure interaction and chromatin loops, together with an observed decrease in expressed genes and active chromatin modifications (Fig. 8e). At different scales, 91.69% and 83.53% of the genome covered by the A/B compartment and TAD-like structure respectively, as well as 23.36% of loops, were stable during fiber development. At each stage, 81.38–91.12% of loops related to homoeologous regions were divergent between the At and Dt subgenomes, serving as a topology for transcriptional regulation such as for expression bias of homoeologous genes (Additional file 2: Fig. S35).

## Discussion

The molecular mechanisms of plant cell differentiation and development have been extensively studied, especially in the model plant *Arabidopsis thaliana*, revealing a complex developmental regulatory network [51, 52]. However, how 3D chromatin organization functions in cell differentiation has been rarely studied in plants. Cotton fiber, as a model system for studying the differentiation of single cells, is regulated by a large number of functional molecular factors [20, 21]. In this study, we used the in situ Hi-C technique to show that the compartment, TAD-like structure, and loop change during fiber development, providing a topological basis for gene transcription. This study establishes a new understanding of the regulatory mechanism underlying the staged differentiation of a typical single cell in plants from the perspective of spatial chromatin interactions.

Genome asymmetry is a common phenomenon observed in interspecific hybrids and allopolyploids, such as cotton, *Brassica*, and wheat [31, 53, 54]. Subgenome dominance reflects the preference of homoeologous gene expression in different subgenomes [55]. Homoeologous gene expression is subject to the regulation of subgenome-specific epigenetic modifications, nearby transposon density, and the frequency of homoeologous chromosome exchanges [56, 57]. In previous studies, genome in situ hybridization (GISH) and Hi-C data showed that the chromosomes of the same subgenome are closer in space [58, 59]. In cotton, the ratio of chromosomal interactions in the At subgenome is higher than in the Dt subgenome, and some genes with biased expression are related to differential chromatin interaction [33]. In this study, we linked the dynamic changes of the 3D structures of the subgenome to gene expression, revealing their regulatory implications in different stages of cotton fiber development. In terms of chromatin compartment, more B compartment switched to A compartment in the At subgenome, while more A compartment switched to B compartment in the Dt subgenome; this compartment switching was correlated with gene expression. From the perspective of TAD-like structure, the At and Dt subgenomes exhibit differential organization because of differential TE activity that partially causes biased expression of homoeologous genes. At the loop level, the At and Dt subgenomes only share a small portion of similar homoeologous interactions and are involved in different regulatory networks. These observations indicate that the two subgenomes are packaged differently and, to a notable extent, contribute coordinately to fiber development, consistent with our previous analysis of the *CesA* gene family [40].

Transposable elements (TE) are recognized as a driver of regulatory renovation of gene expression [60–62]. TE activity can reshape gene regulatory networks in the course of evolution through strategies such as expanding the number and types of binding sites of transcriptional factors [63]. It is also known that transcription of TE can create new TAD boundaries, and the inactivation of TE can eliminate the formation of TAD boundaries [64]. Studies in *Arabidopsis* have found that the expansion of TE affects the compactness of chromatin from different parents in hybrids [65]; heat stress induces TE activation, and these activations are related to changes in local chromosomal interactions [17]. It is found in rice that the H3K9me2-binding sites with higher TE density may be involved in chromatin interaction [66]. During the evolution of the cotton genome, active TE amplification was coupled with the formation of lineage-specific boundaries of TAD-like structure [34]. In the process of fiber development, we found that the expression levels of TE at boundaries are higher than those in interiors, and subgenome-specific boundaries are enriched in active TE, further indicating the topological role of TE in demarcating TAD-like structure in cotton fiber.

*Cis*-regulatory elements, including promoters, enhancers, silencers, and insulators, represent a basis for a proposed functional role of higher-order chromatin structure, and its participation in the regulation of gene expression [67]. The expression of a single gene is usually jointly regulated by multiple *cis*-regulatory elements that can be linked by chromatin loops and *trans*-acting factors [68, 69]. Many studies have identified functional candidates of *cis*-regulatory elements in cell differentiation and developmental stages in plants [70–72]. In this study, we used high-resolution Hi-C data to identify a large number of gene-non-gene loops, and most non-gene regions likely contain a large number of *cis*-regulatory elements remote to target genes. Therefore, beyond functional studies of coding genes, this study paves the way for decoding the functional roles of non-coding genomic sequences in cell differentiation. Further manipulation of the remote bona fide *cis*-regulatory elements will provide new insights into the regulatory mechanisms underpinning these processes.

## Conclusions

In this study, we constructed a dynamic map of spatial chromatin structure in cotton fiber development. We found a general pattern of chromatin status switching from active (A compartment) to inactive (B compartment). A large number of TAD-like structures were reorganized and chromatin loops showed dynamic changes, which showed different trends in the At and Dt subgenomes. This study lays a foundation for further research on the transcriptional regulation of fiber-related genes and informs future cotton fiber improvement. Using cotton fiber as a model, the topological basis of chromatin for staged differentiation of single cells is proposed, which provides a reference for studying the mechanisms of cell differentiation in other plants.

## Methods

### Plant materials

*Gossypium barbadense* 3–79 was planted in the field of Huazhong Agricultural University and was managed regularly in 2018. Cotton plants at the full blooming stage were sampled, and the fibers and ovules were stripped from the cotton ball on the day of flowering (0 day post anthesis, 0 DPA), 5 DPA, 10 DPA, and 20 DPA. Ovule and fiber samples were immediately put into liquid nitrogen for quick freezing and stored in an ultra-low temperature refrigerator at − 80°C for later use.

### RNA-Seq and data analysis

Total RNA from ovule and fiber samples was isolated using a previously described method [73]. RNA-Seq libraries were constructed using the Illumina TruSeq Stranded RNA Kit (Illumina, San Diego, CA, USA) and were sequenced on the MGI2000 system. For each sample, three biological replicates were performed. The clean RNA-Seq reads were mapped to the reference genome of *G. barbadense* using HISAT2 (version 2.1.0) with default settings [41, 74]. High-quality mapping reads (-q 25) were used to calculate gene expression levels using StringTie (version 2.1.4) with parameter settings (--fr -e -G) [75]. In this study, expressed genes were defined as those with FPKM (fragments per kilobase of transcript per million mapped reads) values larger than one in three biological replicates.

### Genomic synteny and homoeologous gene analysis

The MCScanX software was used to identify syntenic blocks between the At and Dt subgenomes [41, 76], with an all-vs-all blastp analysis of all genes (-e 1e-10, -v 5, -b 5). Syntenic blocks were defined as those with at least five syntenic genes. To identify homoeologous genes between the At and Dt subgenomes, a reciprocal all-vs-all blastn analysis was performed for each gene in the At or Dt subgenome using the coding sequences (-e 1e-10, -v 1, -b 1). The best match of each gene in the other subgenome was retained, and only those contained in syntenic blocks were regarded as homoeologous gene pairs.

### ChIP-Seq and data analysis

Chromatin immunoprecipitation was performed as described previously [29]. In this study, three antibodies were used, including H3K27ac (Millipore, 07-360), H3K4me3 (Abcam; ab8580), and H3K9me2 (Abcam; ab1220). Each experiment was performed with two biological replicates. ChIP libraries were constructed using the Illumina TruSeq Sample Prep Kit following the manufacturer’s recommendations and sequenced on the MGI2000 system. The clean sequencing reads were mapped to the *G. barbadense* reference genome using Bowtie2 (version 2.2.4) with parameter settings (-N 1 -L 30) [41, 77]. After discarding PCR duplicates, high-quality mapping reads (-q 25) were subject to peak calling using the MACS software (version 2.2.7.1) (-q 0.01) [78]. The input DNA sequencing data were used as a control with the parameter settings (“macs2 bdgcmp -t filenam_treat_pileup.bdg -c filename_control_lambda.bdg -o filename_fe.bed -m FE”). The fraction of all mapped reads that fall into peak regions (FRiPs) of H3K27ac (41.9~60.51%), H3K4me3 (60.79~80.69%), and H3K9me2 (24.08~47.37%) imply the quality of ChIP-Seq data are suitable for downstream analysis.

### Hi-C library generation and sequencing

The in situ Hi-C experiment was essentially carried out as described previously [45], with minor modifications. In this study, 300–400 cotton ovules were taken for each sample. The fibers were separated from ovules using tweezers and ground into powder in liquid nitrogen. Fiber powder was fixed in NI buffer containing 1% (v/v) formaldehyde at room temperature for 15 min, and then the glycine was added to the solution to terminate the fixation. The fixed fiber powder was processed using a single layer of miracloth filter membrane and then filtered using a single layer of 20 μm nylon membrane to obtain pure nuclei. The nuclear solution was centrifuged, gently resuspended in NEB 3.1 containing 0.15% SDS, and incubated at 62 °C for 7 min to increase the nuclear membrane permeability. After digestion using the DpnII restriction endonuclease, biotin labeling, ligation, and reverse-crosslinking process, 1–1.5 μg DNA was used for DNA manipulation, including removing end markers, ultrasound, end repair, and fragment screening. The 50 μl of 300–500 bp DNA solution was used for library construction. The Dynabeads MyOne Streptavidin T1 (Invitrogen; 65602) magnetic beads were used to purify the DNA fragments containing biotin, and the VAHTS Universal DNA library construction kit for MGI (vazyme; NDM607) was used to build the library. Each step of the washing process in the library construction process included washing twice with Tween Wash Buffer (TWB) to remove non-specific fragments. Hi-C libraries were sequenced on the MGI2000 system. The Hi-C experiment was performed with two biological replicates. The sequencing depths estimated by raw data were 173× (0 DPA), 193× (5 DPA), 220× (10 DPA), and 183× (20 DPA).

### Hi-C sequence data processing

The Hi-C-Pro pipeline is used to obtain valid Hi-C contact read pairs [79]. Briefly, clean sequencing data were mapped to the *Gossypium barbadense* genome using Bowtie2, and multiple hits, low MAPQ, singleton, dangling end, self-circle, and PCR duplicates were removed. An iterative correction and eigenvector decomposition (ICE) method was used to normalize the Hi-C contact matrices at resolutions of 5, 10, 20, 40, and 200 kb. Hi-C matrices were used in downstream analyses.

### Heatmap of interaction frequency and visualization

We used the chromatin interaction matrices generated by HiC-Pro in combination with HiCPlotter and Juicebox to map chromatin interactions in heatmap [79–81]. The Hi-C chromatin interactions were divided into short-range interactions (≤ 2 Mb) and long-range interactions (> 2 Mb) based on the distance of chromatin on the one-dimensional space. The chromatin compactness is calculated by counting reads that can undergo chromatin interactions between each bin and the surrounding 1 Mb range at the 10 kb resolution.

### Contact probability analysis

Contact strength is a term that represents the probability of interactions between two bins [82]. We calculated the bin-bin contact strength (log_10_(sum of observed reads bin-bin in fixed gaps/average reads)) at the 10 kb resolution, and the distance between two bins was controlled within 10 kb–100 Mb.

### Analysis of A and B compartments

The hicPCA program embedded in HiCExplorer was used to delineate A/B compartments at the 40 kb resolution [83]. Bins with the first principal component values greater than zero and having a high gene density on the chromosome level were regarded as A compartment; bins with values less than zero and having a low gene density were regarded as B compartment. If the values of at least two consecutive bins changed from positive to negative, these bins represented switching from A compartment to B compartment, and vice versa represented switching from B compartment to A compartment.

### Analysis of topologically associated domain-like structure (TAD-like structure)

TAD-like structures were identified using the hicFindTADs program embedded in HiCExplorer at the 20 kb resolution [83]. The insulation score in a bedgraph file was also calculated using this program. Conserved TAD-like structure boundaries between two developmental stages were defined as those with a maximum boundary change of two resolution distance (40 kb). Conserved boundaries were defined as those with two conserved boundaries. Homoeologous TAD-like structures between two subgenomes were defined as those which contained precisely the same homoeologous genes. Partitioned TAD-like structure contained inconsistent subgenomic genes. In addition, the TADCompare software with default settings [84] was used to analyze dynamic TAD-like structure in fiber development, serving as a comparison to the above method. The observation that ~ 76.98% of TADs were conserved between two adjacent periods identified by TADCompare was largely consistent with the result of the first method (~ 74.4%) (Additional file 14: Table S13).

### Identification of TAD-like structure cliques

TAD clique is a fully connected network with TAD and TAD interaction, and each TAD in this network can interact with some other TADs [43]. The size of the fully connected network indicates the size of TAD cliques. We used the connection strength of loops between two TAD-like structures to measure whether two TAD-like structure had interaction:

$$S=\frac{B_1\times {B}_2}{L}$$

*S* represents the interaction strength between two TAD-like structure, *B* refers to the bin number of TAD-like structure, and *L* stands for the number of interaction loops between them. This model eliminates the effect of structure size differences. Since clique is a larger-scale spatial structure, we considered more loops involved in the interactions between TAD-like structures (Contact count > 5 and false discovery rate (FDR) < 0.1). In order to define the interactions between TAD-like structures accurately, we used a threshold value (*S* > 0.09) to filter out some low-intensity interactions.

### Analysis of chromatin loops

In this study, FitHiC2 was used to identify chromatin loops at the 5 kb resolution (FDR < 0.005 and contactCount > 10) [85]. To avoid the influence of length difference of chromatin between gene-poor regions (gene number ≤ 20 in 500 kb) and gene-rich regions (gene number > 20 in 500 kb), the proportion of loop anchors were normalized by chromatin length. Likewise, the loop density in chromosomes was normalized by the number of loops at different stages.

### Construction of the regulatory network

We constructed homoeologous gene regulatory networks in the At and Dt subgenomes using genes as nodes and gene-related loops as edges. Networks were constructed using Cytoscape with the edge-weighted spring embedded layout method based on a “force-directed” paradigm as implemented [86]. Homoeologous genes are placed in identical positions in the networks. However, the size of the nodes differs due to the difference in the number of loops related to homoeologous genes. In other words, the size of a node is positively correlated with the number of loops that are connected. There were some loops located in non-genic regions, which might play a sustaining role and were discarded. We defined two different types of homoeologous loops: gene-gene loop (G-G loop) and gene-non-gene loop (G-N loop). Similar G-G loops between two subgenomes represented those in which both loop anchors were located in homoeologous gene regions. Similar G-N loops represented those with one anchor located in a homoeologous gene region.

### Statistical analysis

Differentially expressed genes were identified using the DESeq2 package in R with the negative binomial distribution, and only those with expression fold change log2(fold_change) > 1 and FDR < 0.01 were retained [87]. Gene Ontology (GO) enrichment analysis was carried out using a two-sided Fisher’s exact test, and GO terms with a FDR of less than 0.05 were retained.

## Supplementary Information

**Additional file 1: Table S1.** Summary of RNA-seq and ChIP-seq raw data.

**Additional file 2: Figures S1–S35.**

**Additional file 3: Table S2.** Summary of differentially expressed genes in fiber development.

**Additional file 4: Table S3.** Summary of homoeologous genes with expression bias in fiber development.

**Additional file 5: Table S4.** GO enrichment for different clusters.

**Additional file 6: Table S5.** Summary of Hi-C data in this study.

**Additional file 7: Table S6.** Genes included in the compartment switching regions.

**Additional file 8: Table S7.** The GO terms enriched by genes contained in the compartment switching regions.

**Additional file 9: Table S8.** TAD-like structure regions during fiber development.

**Additional file 10: Table S9.** Genes contained in dynamic TAD-like structure boundaries.

**Additional file 11: Table S10.** Homoeologous TAD-like structures in fiber development.

**Additional file 12: Table S11.** Partitioned TAD-like structures in fiber development.

**Additional file 13: Table S12.** TAD-like structures that form cliques in fiber development.

**Additional file 14: Table S13.** Conserved or differential TAD-like structure boundaries calculated by TADCompare.

**Additional file 15.** Review history.

## Data Availability

All the raw sequencing data generated during the current study are available in the NCBI BioProject database under accession number PRJNA748268 (https://www.ncbi.nlm.nih.gov/bioproject/PRJNA748268) [88].
