# Supplementary material for: Dynamic 3D genome architecture of cotton fiber reveals subgenome-coordinated chromatin topology for 4-staged single-cell differentiation
Source: Genome Biol. 2022 Feb 3;23:45. doi: 10.1186/s13059-022-02616-y (PMC8812185; doi:10.1186/s13059-022-02616-y)
Supplement: Supplementary file 2 — Additional file 2: Figures S1–S35. [file 13059_2022_2616_MOESM2_ESM.pdf]

Dynamic 3D genome architecture of cotton fiber reveals subgenome-coordinated chromatin topology for 4-staged single-cell differentiation

Liuling Pei[1,4], Xianhui Huang[1,4], Zhenping Liu[1], Xuehan Tian[1], Jiaqi You[1], Jianying Li[1], David D. Fang[2], Keith Lindsey[3], Longfu Zhu[1], Xianlong Zhang[1] & Maojun Wang[1*]

[1]National Key Laboratory of Crop Genetic Improvement, Hubei Hongshan Laboratory, Huazhong Agricultural University, Wuhan 430070, Hubei, China.

[2]Cotton Fiber Bioscience Research Unit, USDA-ARS, Southern Regional Research Center, New Orleans, LA 70124, USA.

[3]Department of Biosciences, Durham University, Durham DH1 3LE, United Kingdom.

[4]These authors contributed equally to this work.

*Correspondence should be addressed to M.W. (mjwang@mail.hzau.edu.cn).

Supplementary Figures

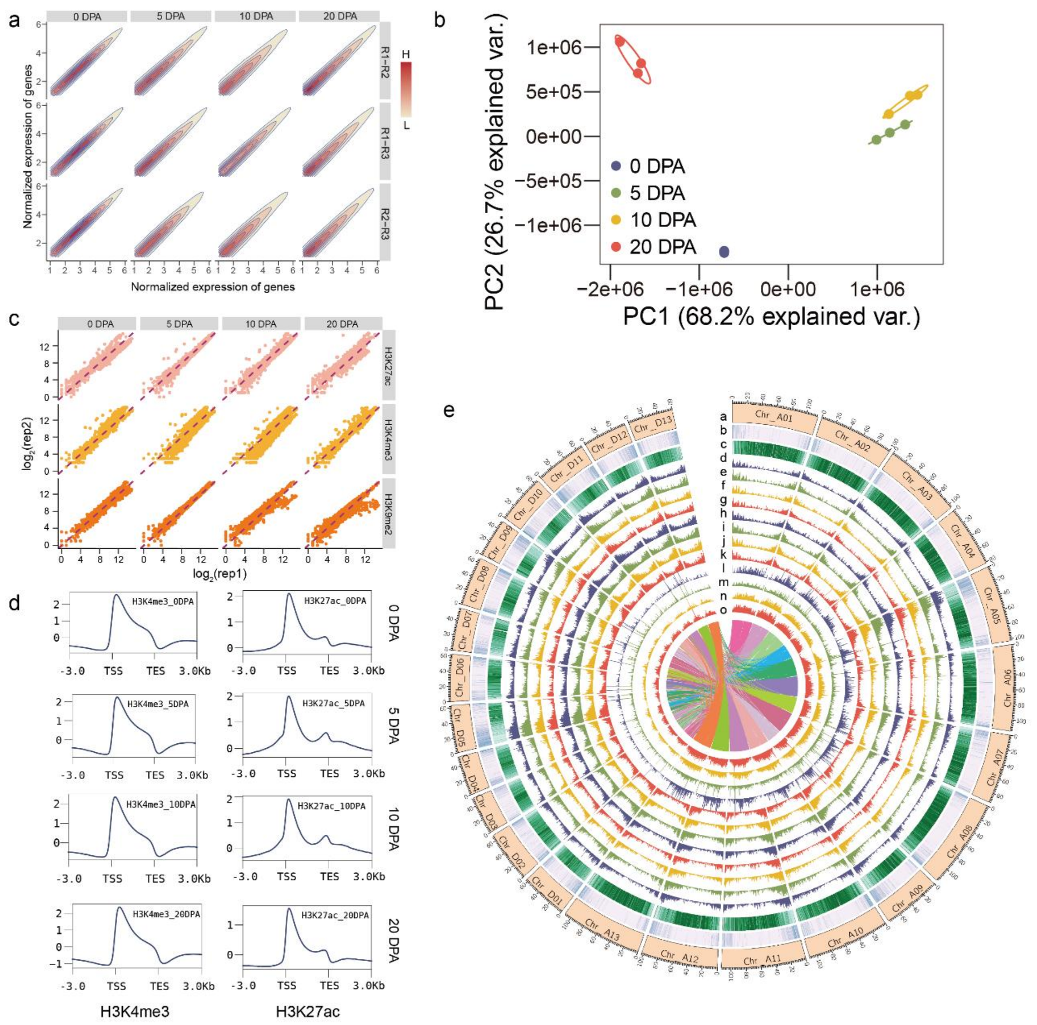

Fig. S1

Reproducibility analysis of RNA-Seq and ChIP-Seq data.

a, The comparison of the expression level of genes between two biological replicates of RNA-Seq data. High density of genes is shown in red. **b**, The PCA analysis of RNA-Seq data with three biological replicates in four periods during fiber development. **c**, The dot plots show the consistency of two biological replicates of ChIP-Seq data. This analysis is performed in 100-Kb windows. **d**, Distribution of H3K4me3 and H3K27ac signals around 3 Kb in TSS and TES during fiber development. **e**, The circos plot showing the distribution of gene density, TE density, H3K4me3, H3K27ac, H3K9me2 peaks on a genome-wide scale and contact of homoeologous genes. a-o, Circos plot from outer to inner tracks showing chromosome (a), gene density (b), TE density (c), d-g represent the H3K27ac peaks from 0 DPA to 20 DPA, h-k represent the H3K4me3 peaks from 0 DPA to 20 DPA, l-o represent the H3K9me2 peaks from 0 DPA to 20 DPA.

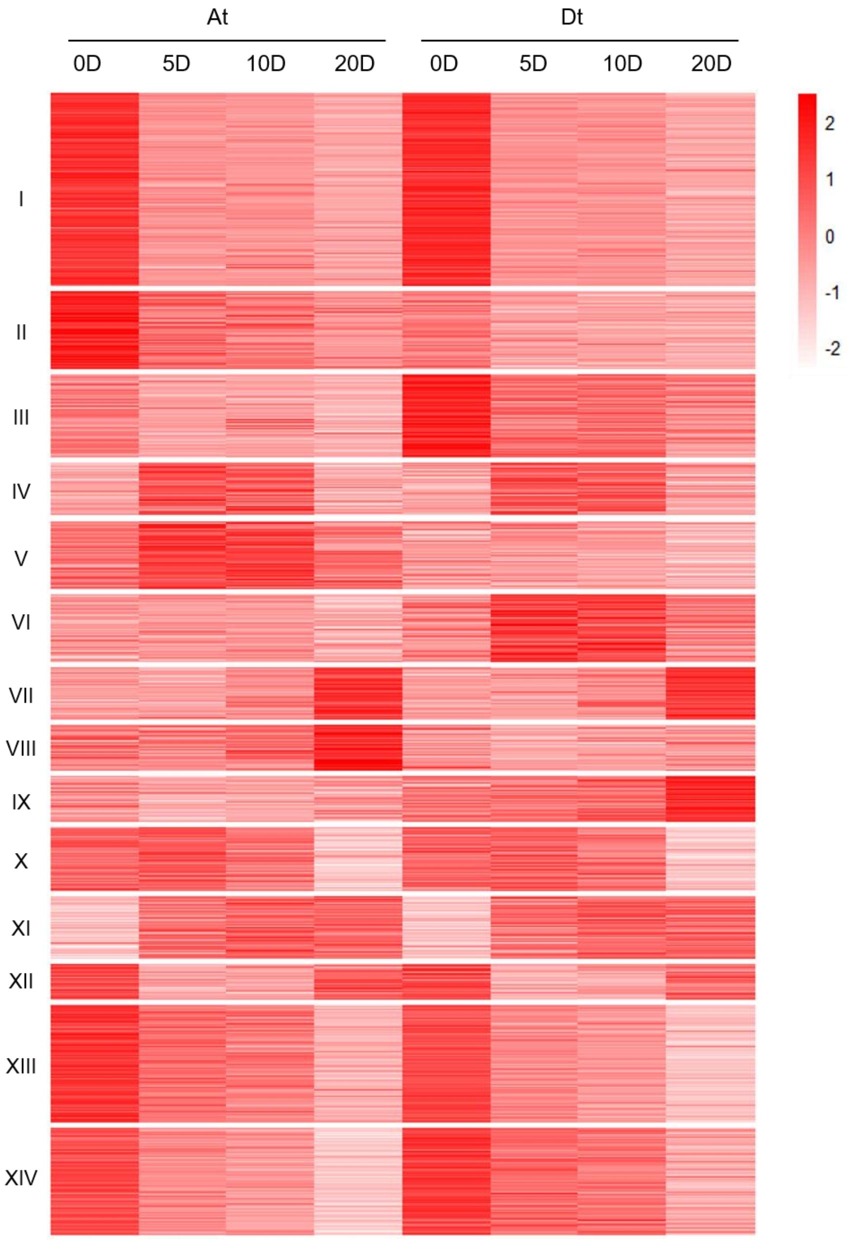

Fig. S2

K-means clustering of the expression patterns of homoeologous genes in four developmental stages. In this analysis, 19,506 homoeologous genes with expression in at least one stage were included.

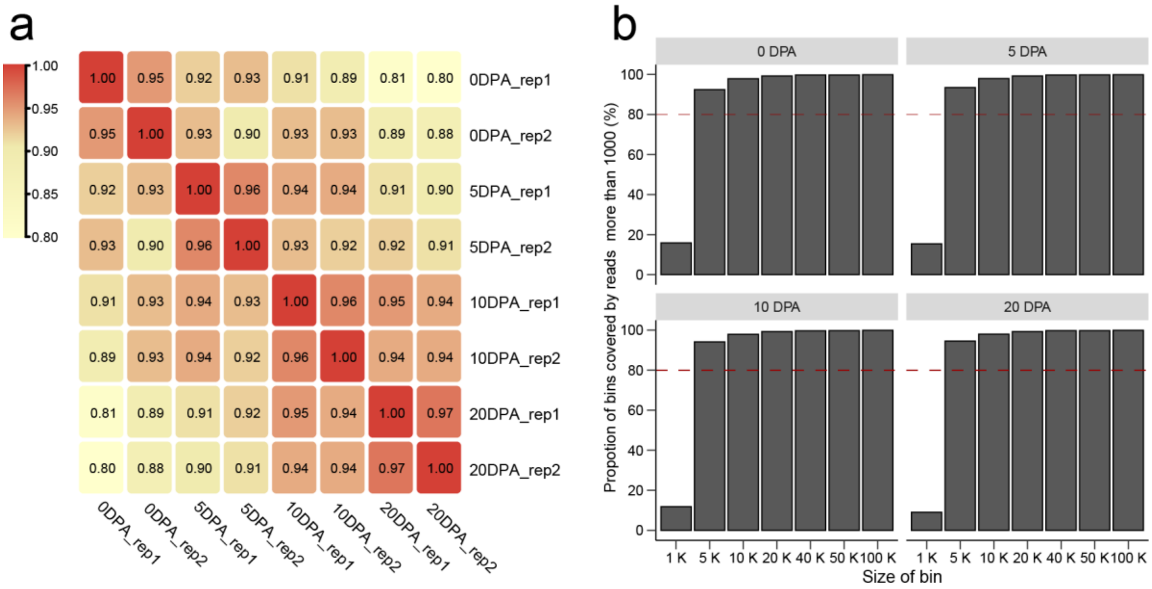

Fig. S3

Reproducibility and resolution analysis of Hi-C data.

a, The heatmap plot shows the correlation coefficient between biological replicate samples at 0 DPA, 5 DPA, 10 DPA and 20 DPA. This analysis was performed using HiCRep software at the 40 Kb resolution. b, The bar plot shows the proportion of bins that contained over 1000 contact reads at different resolutions (1 Kb, 5 Kb, 10 Kb, 20 Kb, 40 Kb, 50 Kb, 100 Kb) during fiber development.

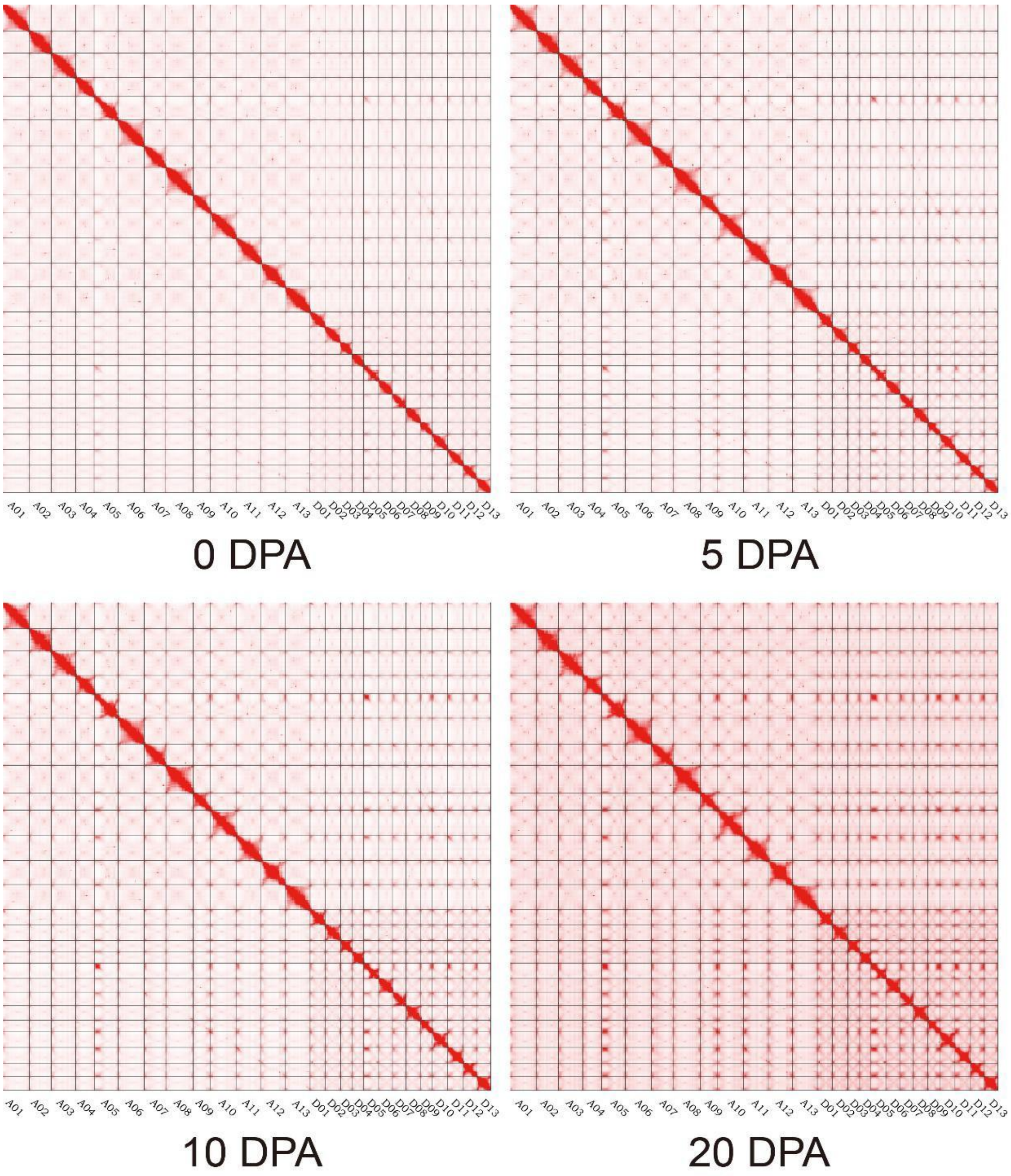

Fig. S4

The heatmap shows chromatin interactions at the chromosome level. The heatmap was constructed at a resolution of 100 Kb.

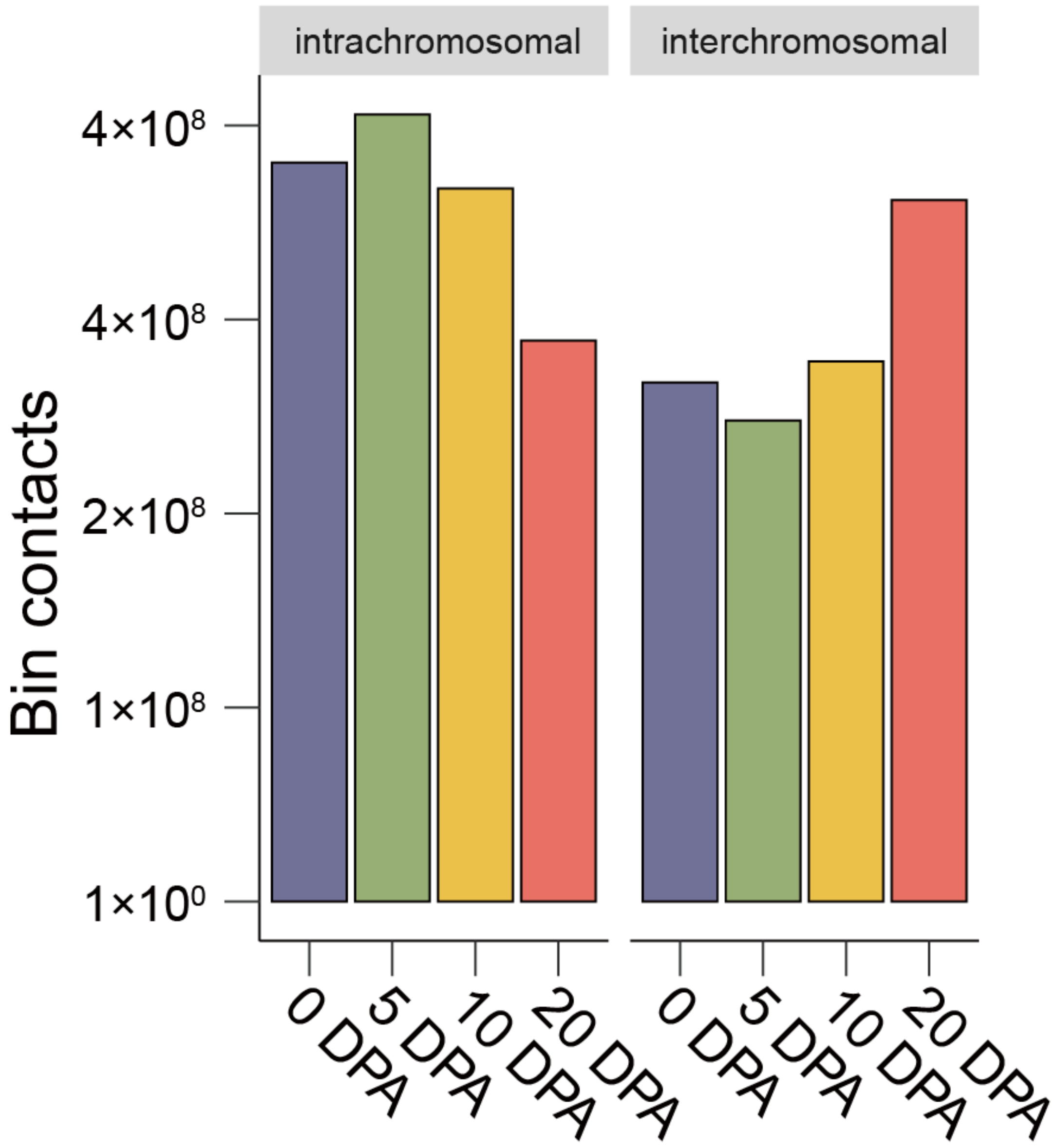

Fig. S5

The bar plot shows the number of intrachromosomal/interchromosomal interactions. In this analysis, Hi-C matrices with a resolution of 10 Kb were used.

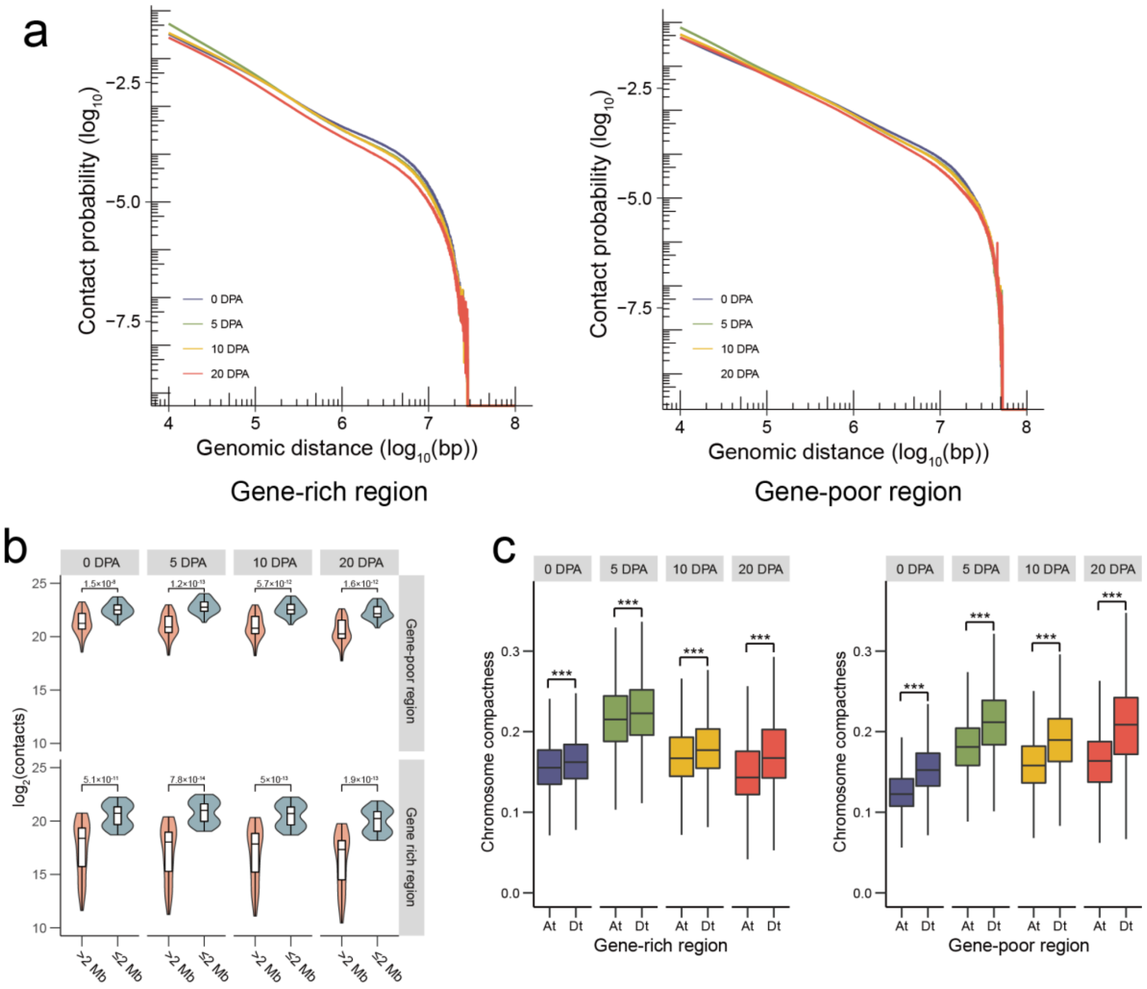

Fig. S6

Analysis of chromatin interactions in gene-rich region and gene-poor region.

a, The line plot shows the chromatin contact probabilities. **b,** The violin plot shows the short-range interaction reads (⩽2 Mb) and the long-range interaction reads (>2 Mb). **c,** Box plot showing the chromosome compactness. For both **b** and **c**, two-sided Wilcoxon signed-rank test was used (***$P < 2.2 \times 10^{-16}$).

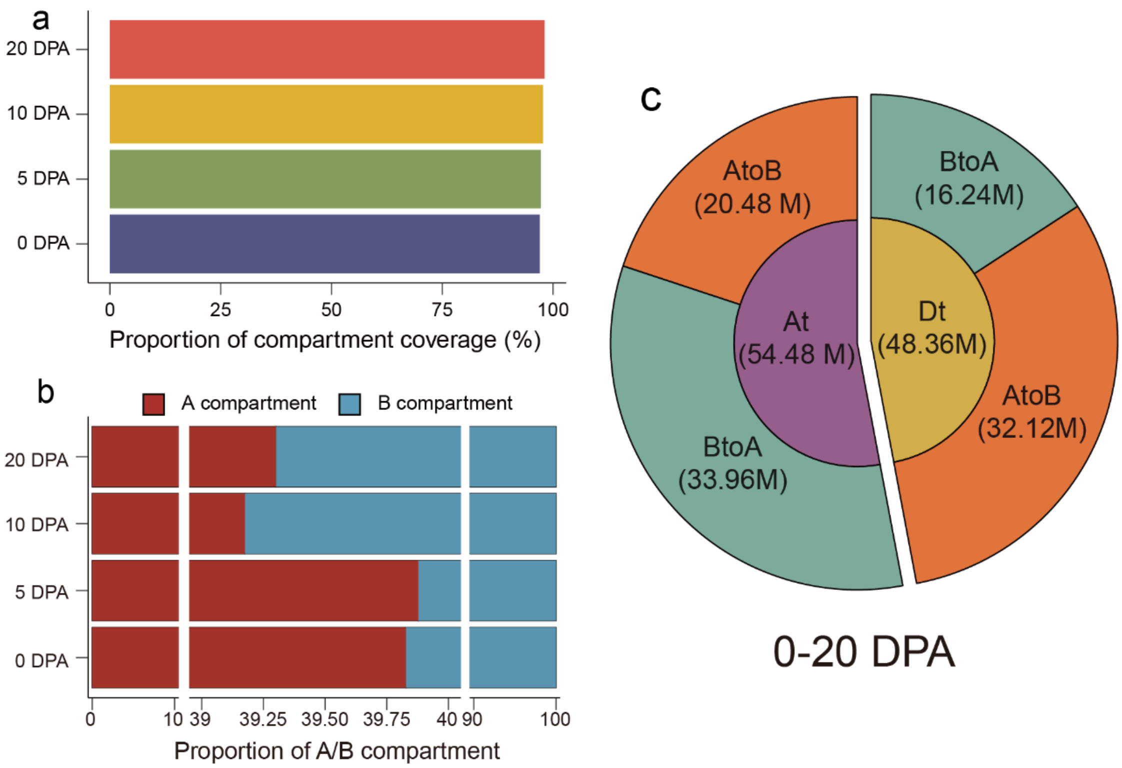

Fig. S7

Genome coverage of compartment and dynamics of A/B compartment during fiber development.

a, The bar plot showing the proportion of genomic length covered by A/B compartment at four stages. **b,** The bar plot depicted the proportion of genomic length categorized as A/B compartment at four stages. **c,** The pie chart showing the switching sizes of A/B compartment in the At and Dt subgenomes.

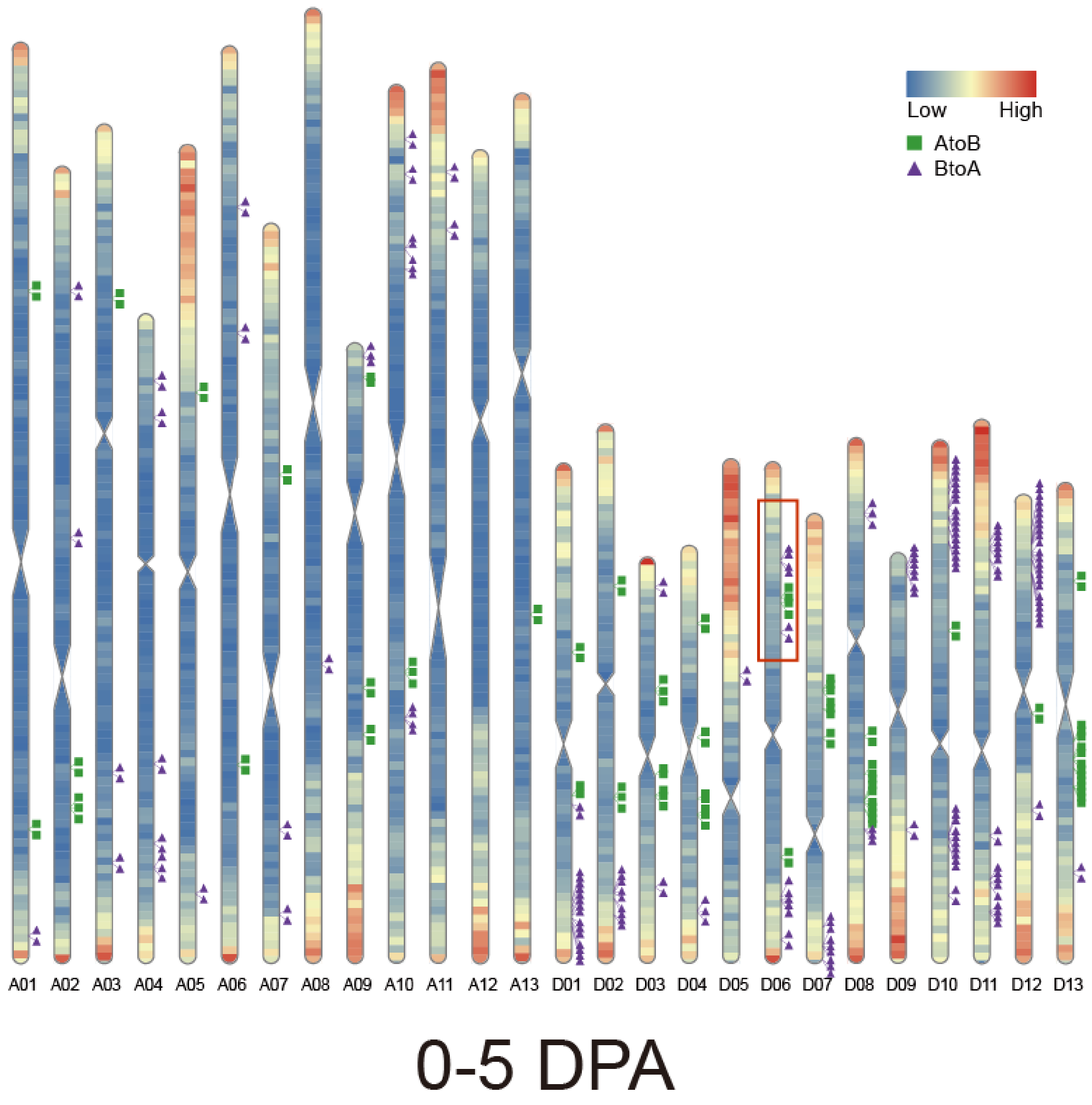

Fig. S8

The positions of the A/B compartment switching regions from 0 DPA to 5 DPA in all chromosomes. The color in each chromosome indicates the gene density. This figure was plotted using an R package Rideogram.

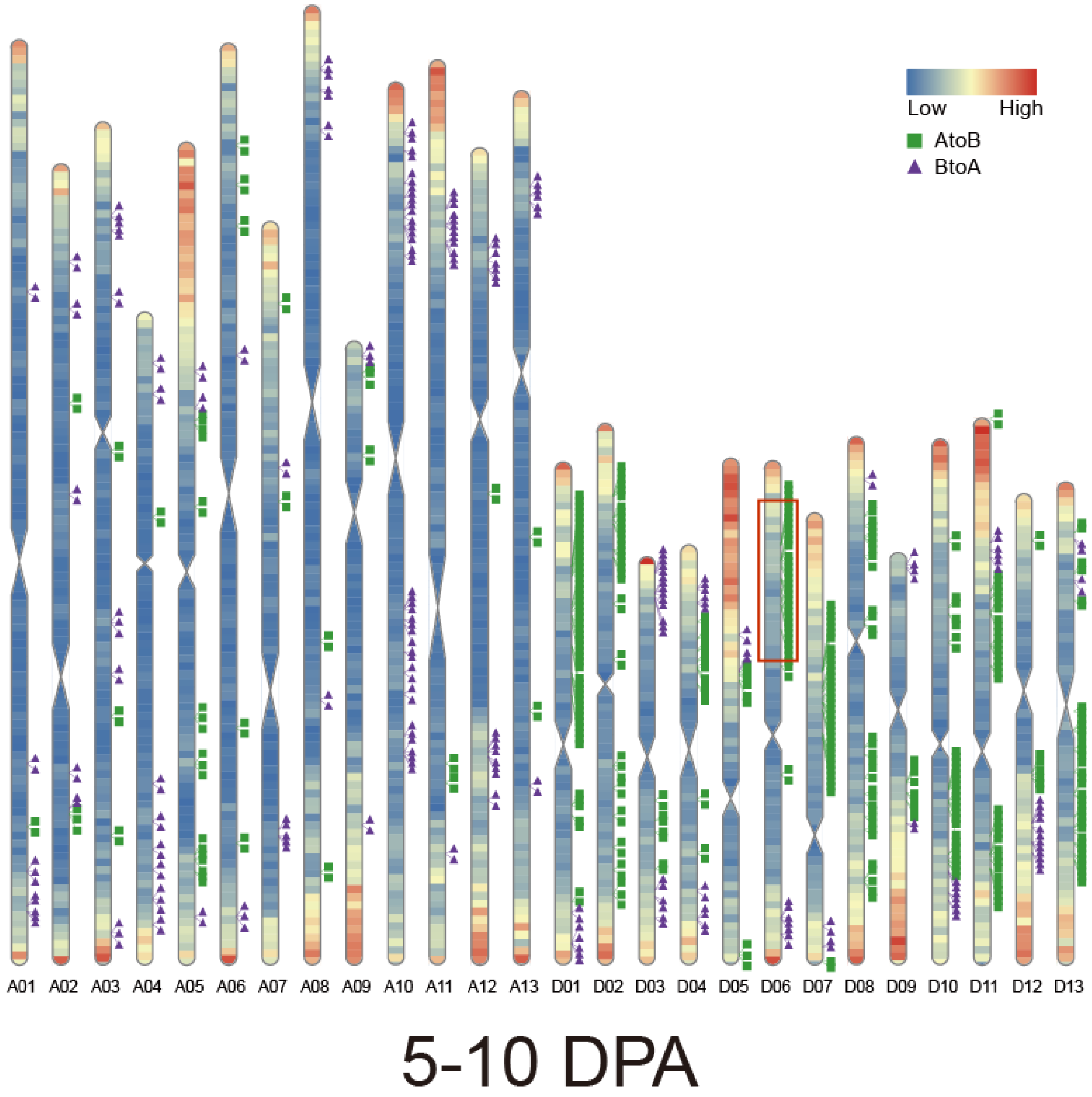

Fig. S9

The positions of the A/B compartment switching regions from 5 DPA to 10 DPA in all chromosomes. The color in each chromosome indicates the gene density. This figure was plotted using an R package Rideogram.

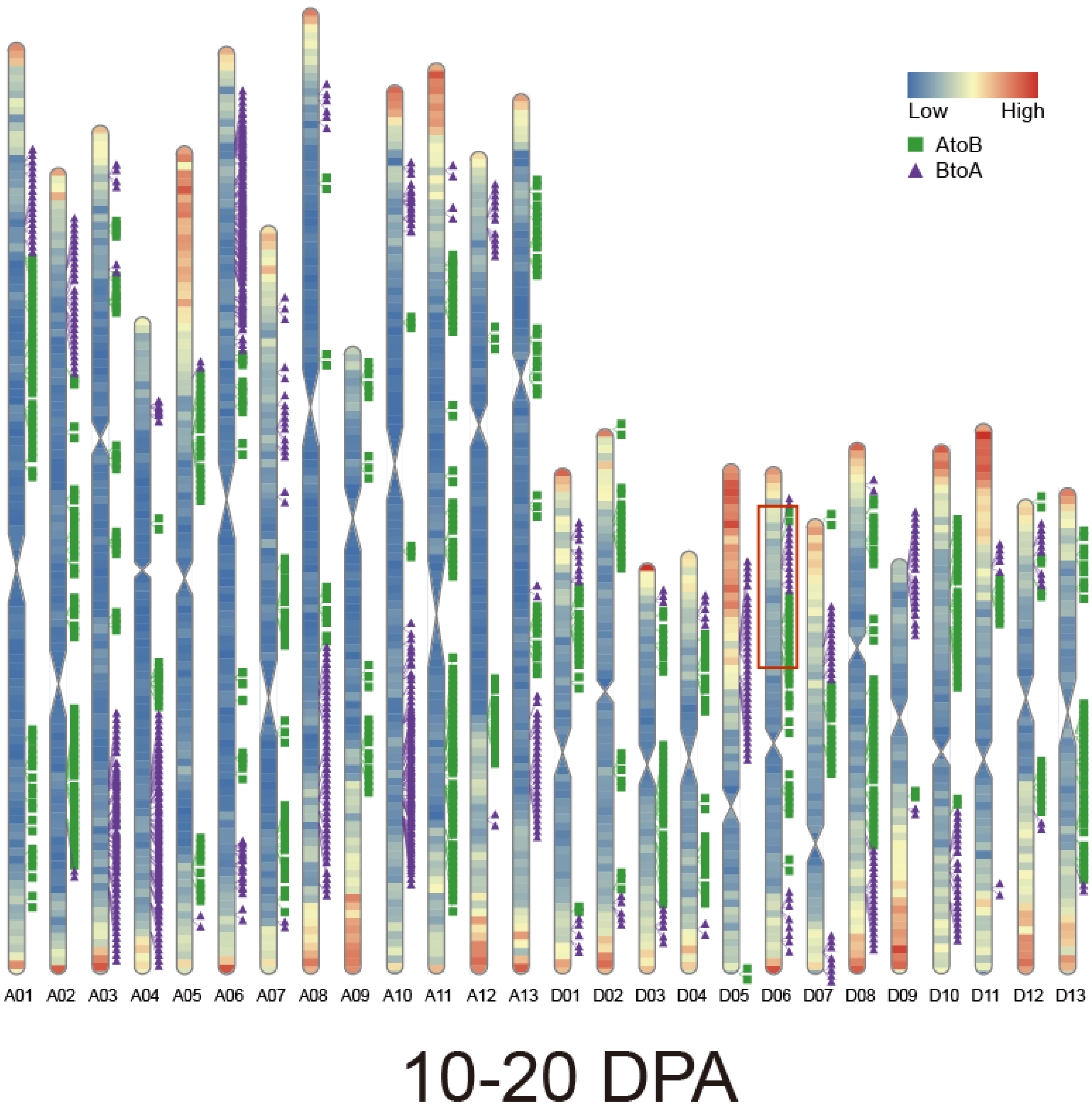

Fig. S10

The positions of the A/B compartment switching regions from 10 DPA to 20 DPA in all chromosomes. The color in each chromosome indicates the gene density. This figure was plotted using an R package Rideogram.

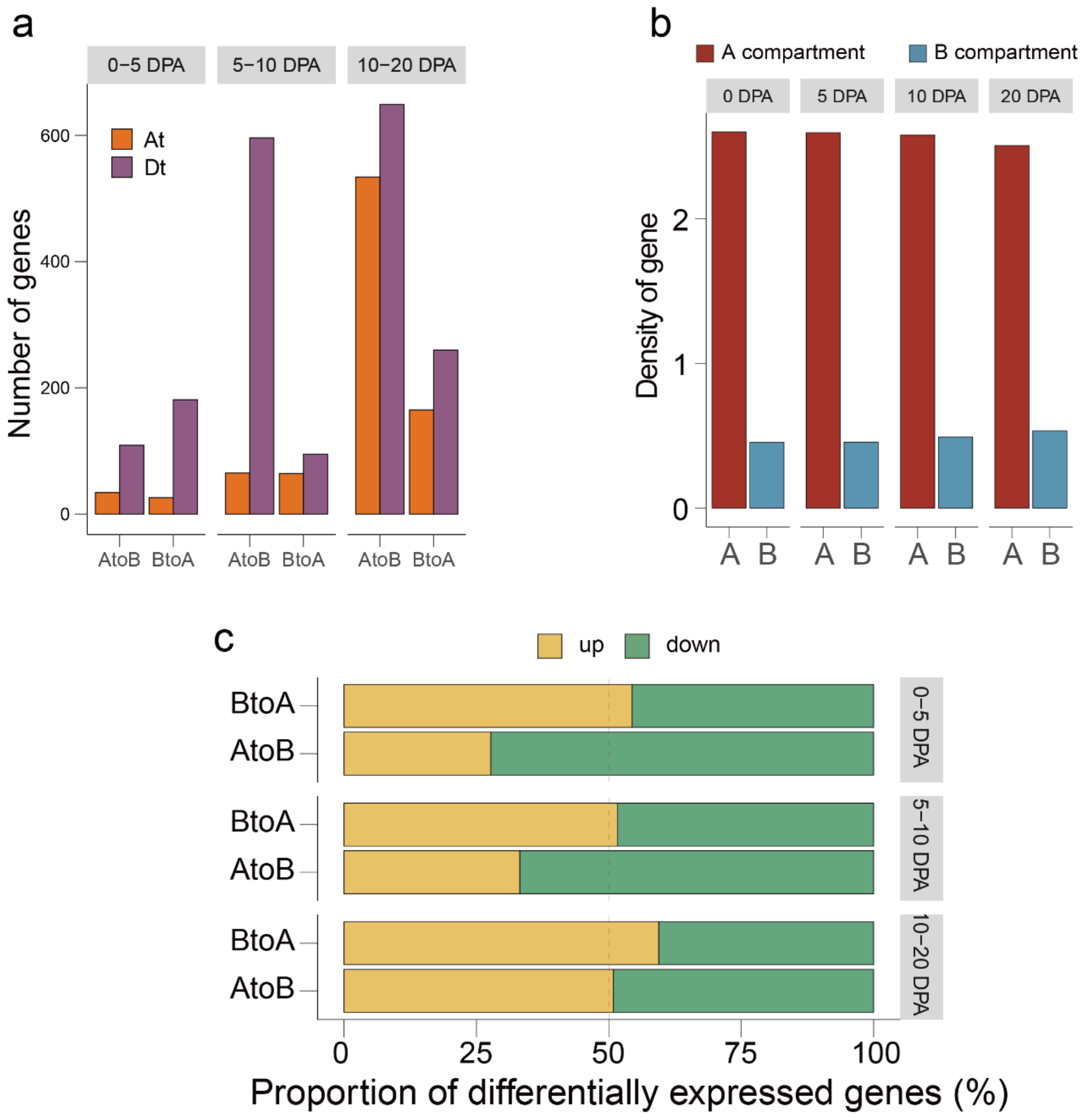

Fig. S11

Analysis of the genes contained in the A/B compartment.

a, The bar plot showing that the number of genes located in two adjacent switching regions. **b,** The bar shows the average number of genes in the A compartment and the B compartment. c, The bar plot shows the proportion of differentially expressed genes contained in switching regions.

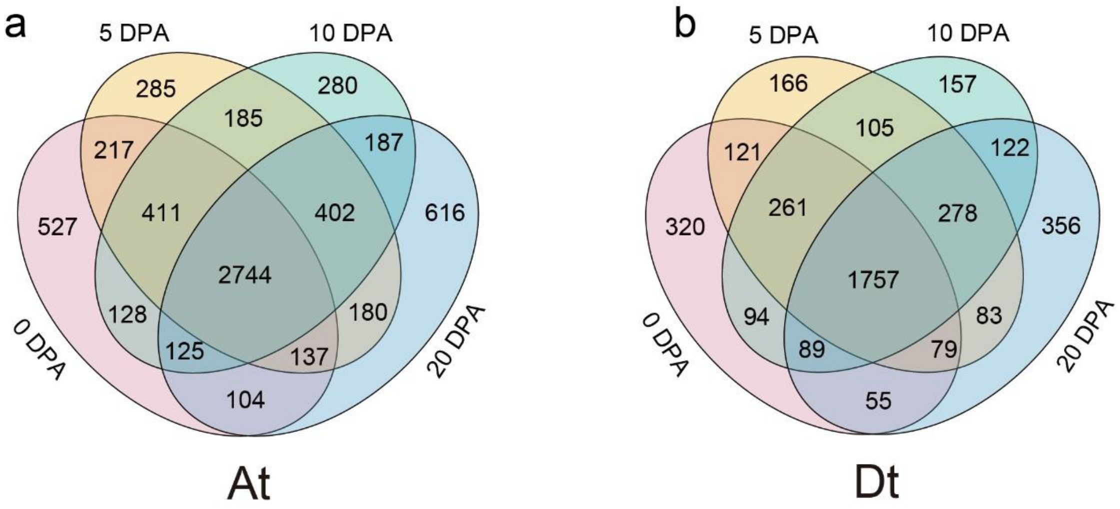

Fig. S12

The number of TAD in the At and Dt subgenomes at four stages.

a, The Venn plot shows the dynamic and conserved number of TADs of the At subgenome at four stages. **b,** The Venn plot shows the dynamic and conserved number of TADs of the Dt subgenome at four stages.

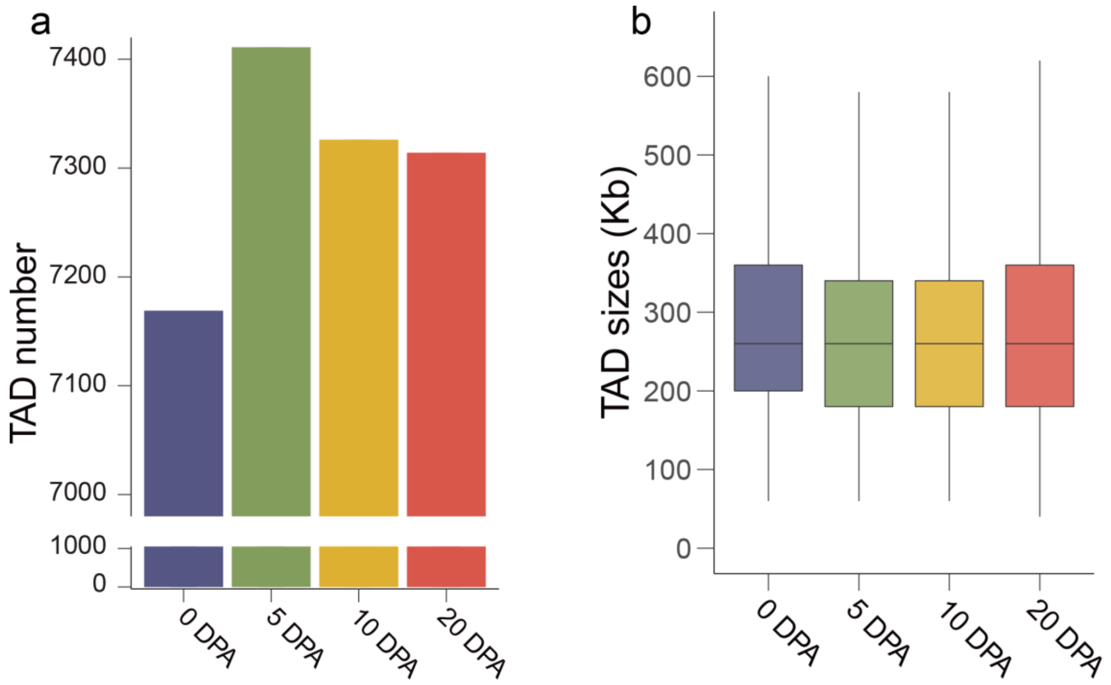

Fig. S13

The number and size of TAD at four stages.

a, The bar plot shows the number of TADs at four stages. **b,** The bar plot shows the size of TADs at four stages.

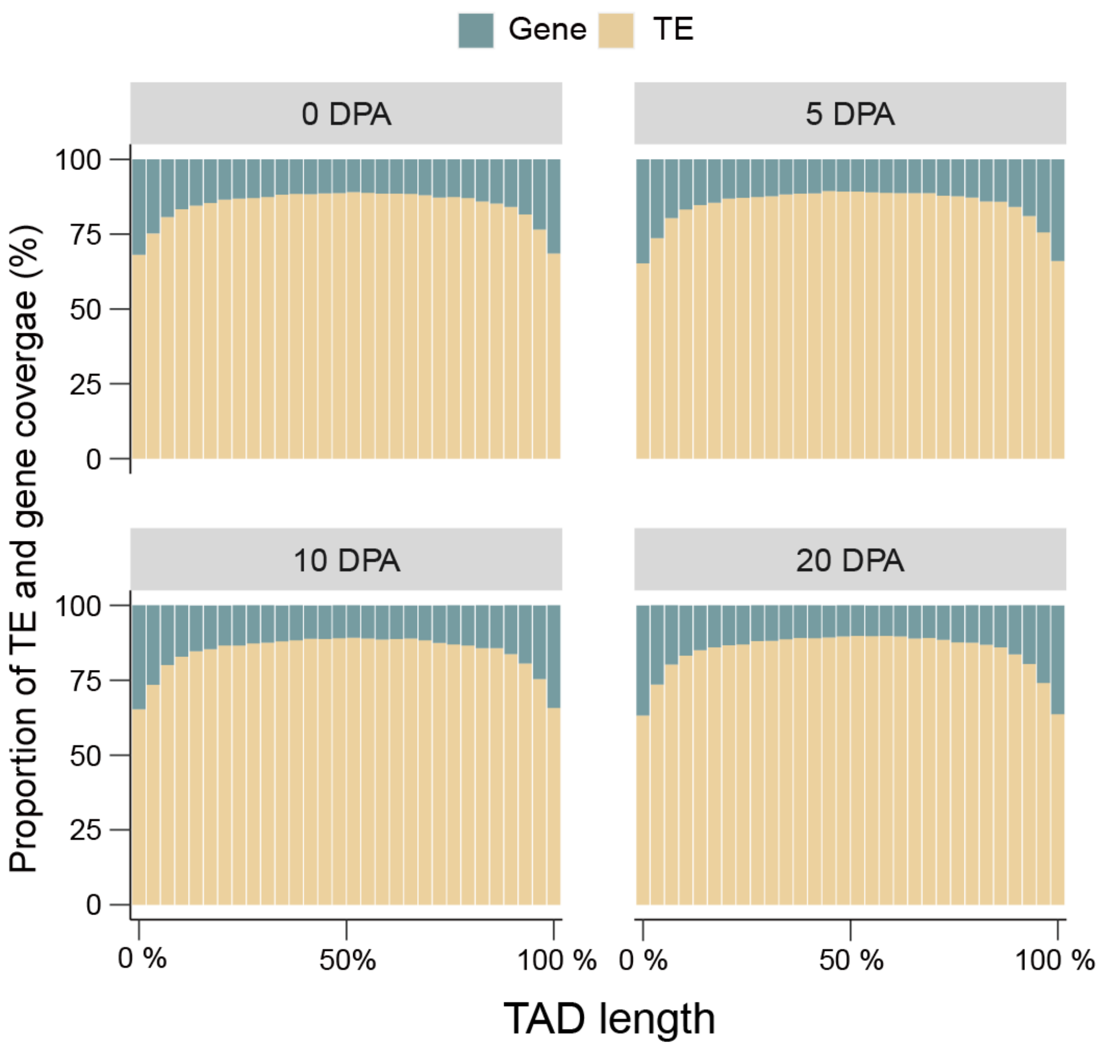

Fig. S14

The coverage of gene and TE in chromosomes during fiber development.

The proportion of TE and gene coverage in TADs during fiber development. We divided each TAD into 30 aliquots and counted the mean values of TE and gene coverage within these regions and analyze the proportion between TE and gene coverage in each region.

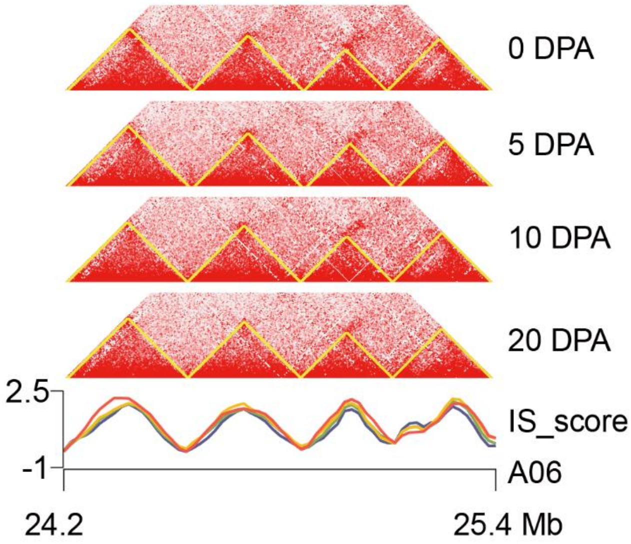

Fig. S15

The heatmap showing the stable TAD architecture in chromosome A06 during fiber development.

The yellow triangle in the heat map represents a TAD-like structure and the wavy line under the heat map indicates the insulation score (0 DPA: Blue; 5 DPA: Green; 10 DPA: Yellow; 20 DPA: Red).

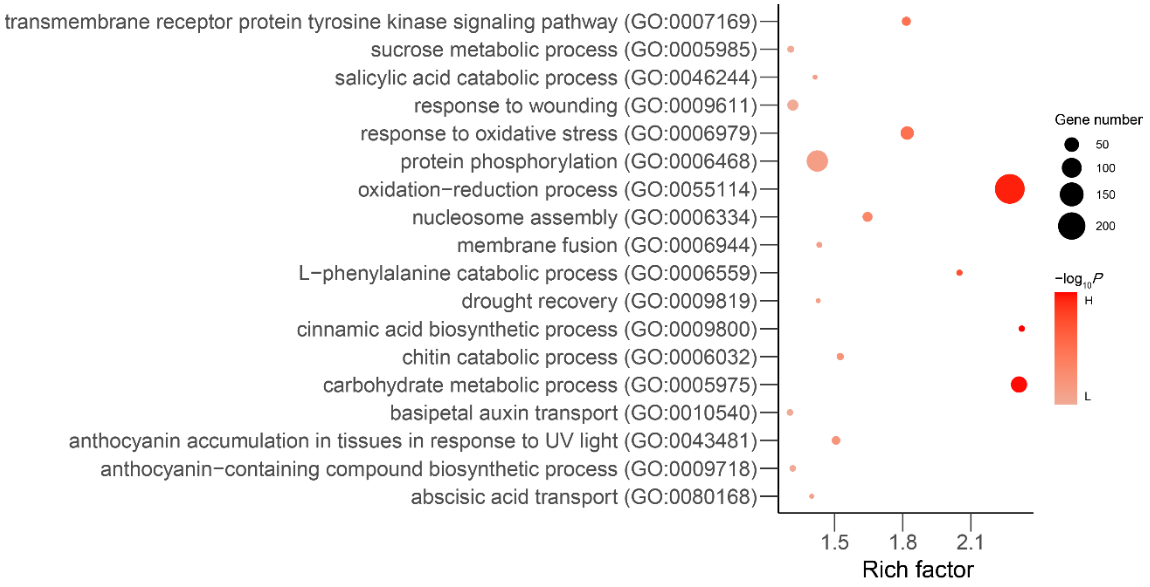

Fig. S16

GO enrichment analysis of differentially expressed genes.

The point plot shows the enriched GO terms of differentially expressed genes located in dynamic TAD boundaries.

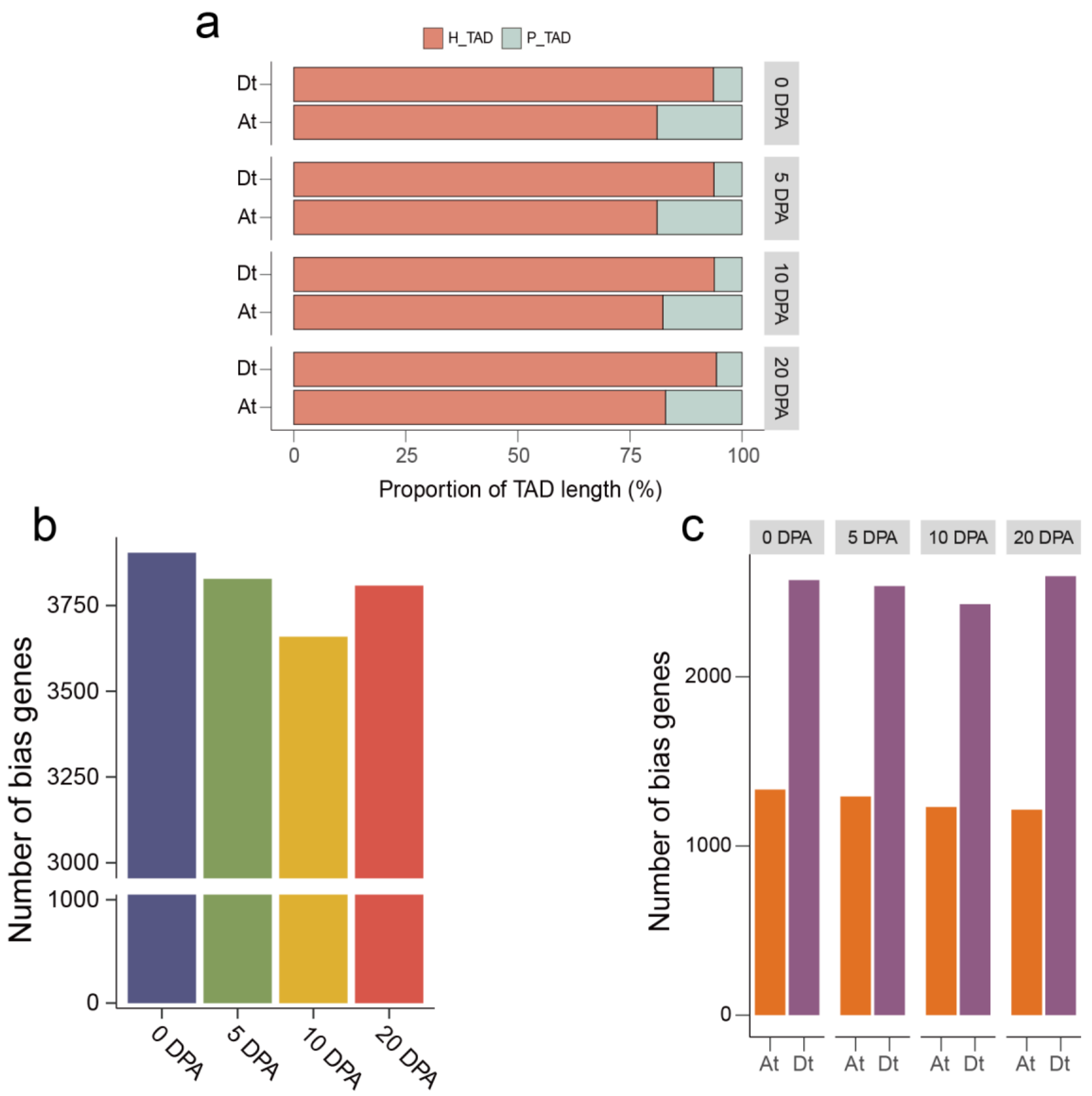

Fig. S17

Analysis of homoeologous TADs and genes with expression bias.

a, The proportion of the length of homoeologous TAD (H_TAD) and partitioned TAD (P_TAD) between the At and Dt subgenomes at four stages. **b,** Bar plot showing the number of homoeologous genes located in TADs with biased expression. **c,** The number of genes with biased expression in the At and Dt subgenomes.

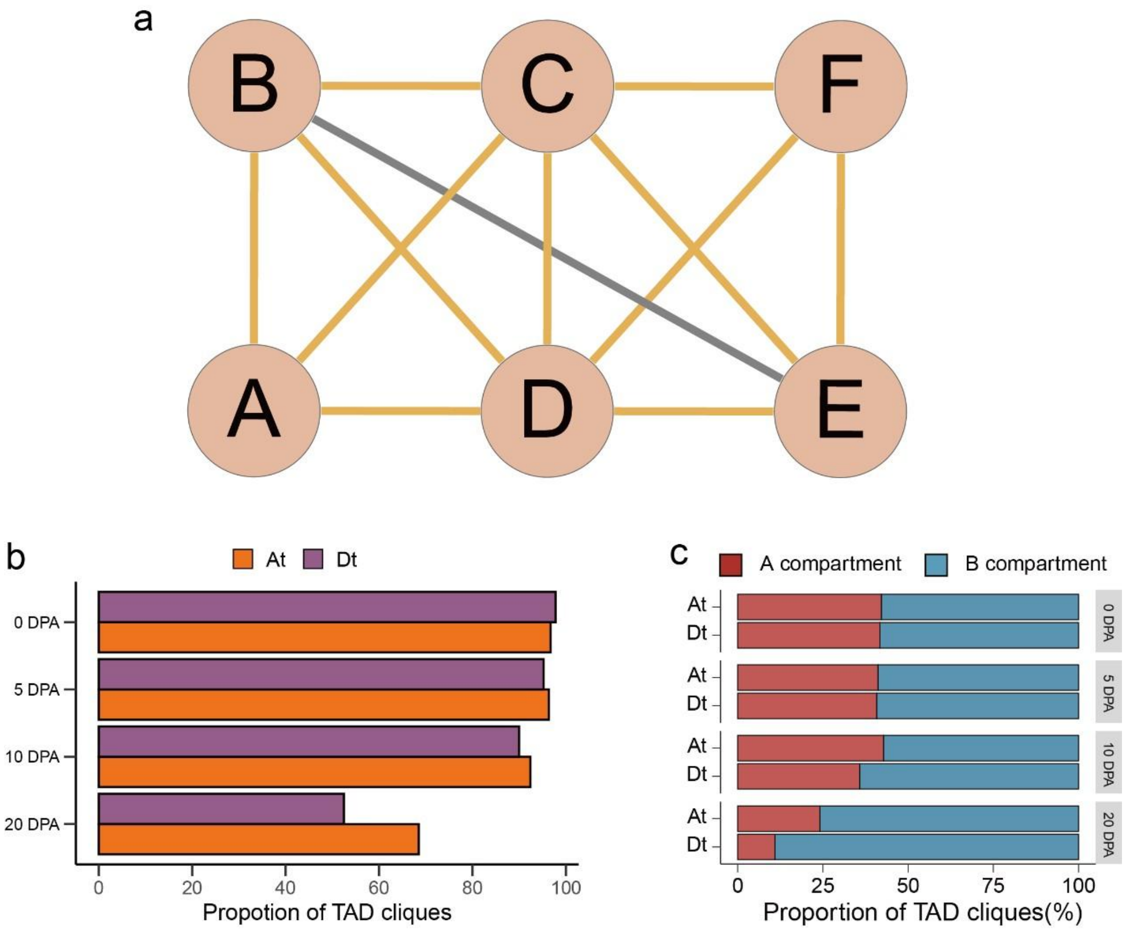

Fig. S18

TAD cliques network model diagram and analysis of TAD cliques.

a, The network plot shows the method for the construction of TADs interaction network. The yellow lines indicate the interactions in TAD cliques. **b,** The bar plot shows the proportion of TAD cliques in the two subgenomes at four stages. **c,** The bar plot shows the proportion of TAD cliques in the A/B compartment at four stages.

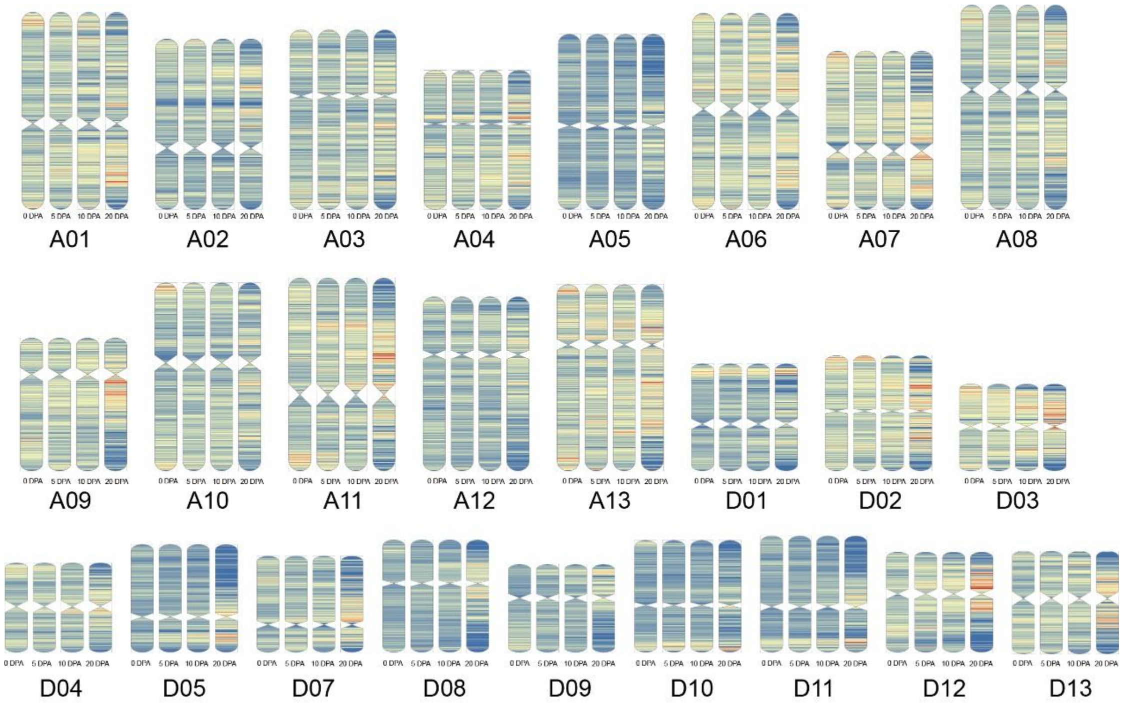

Fig. S19

The TAD interactions in chromosomes during fiber development.

Strong interactions are shown in red and weak interactions are shown in blue.

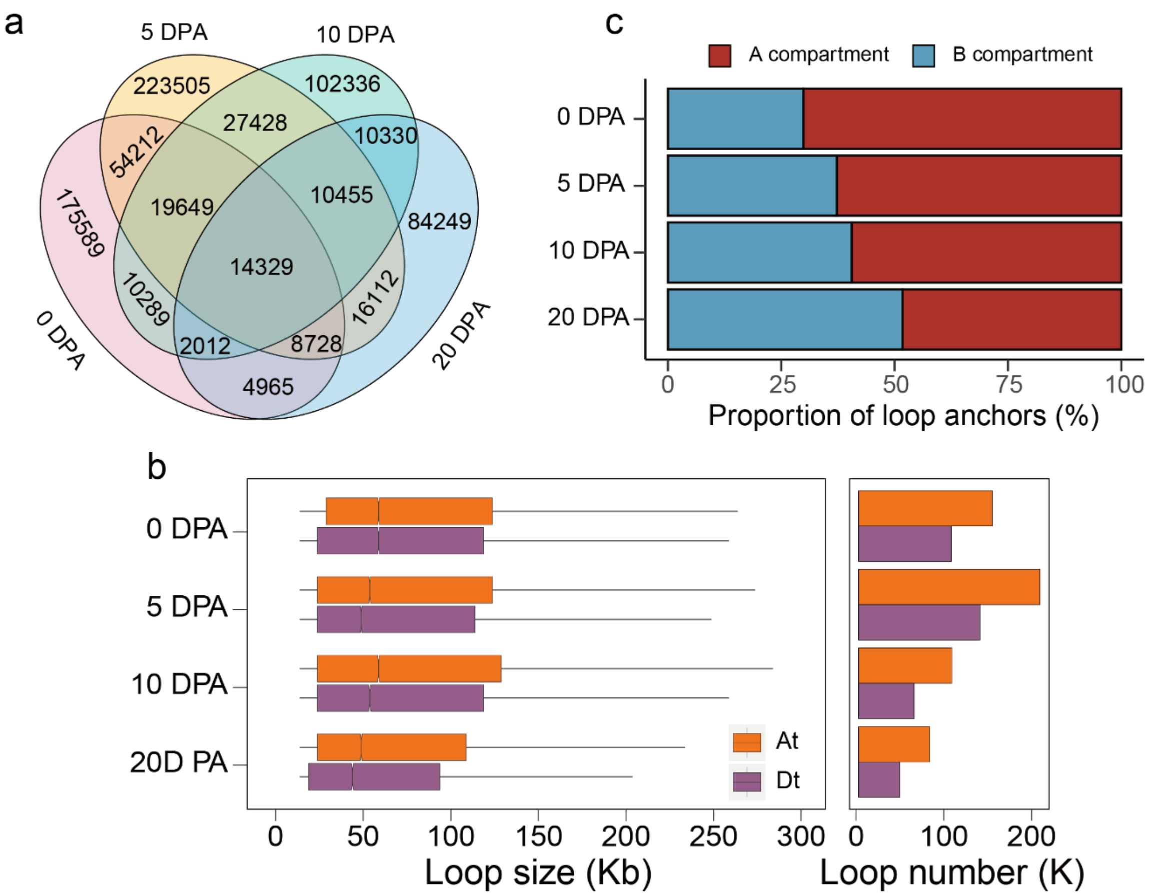

Fig. S20

The number of loops and the distribution of loop anchors on the A/B compartment.

a, The Venn plot shows the dynamic and conserved number of loops at four stages. **b,** Box plot showing the size of loops in the At and Dt subgenomes. Bar plot showing the number of loops. **c,** The bar plot shows the proportion of loop anchors in the A/B compartment at four stages.

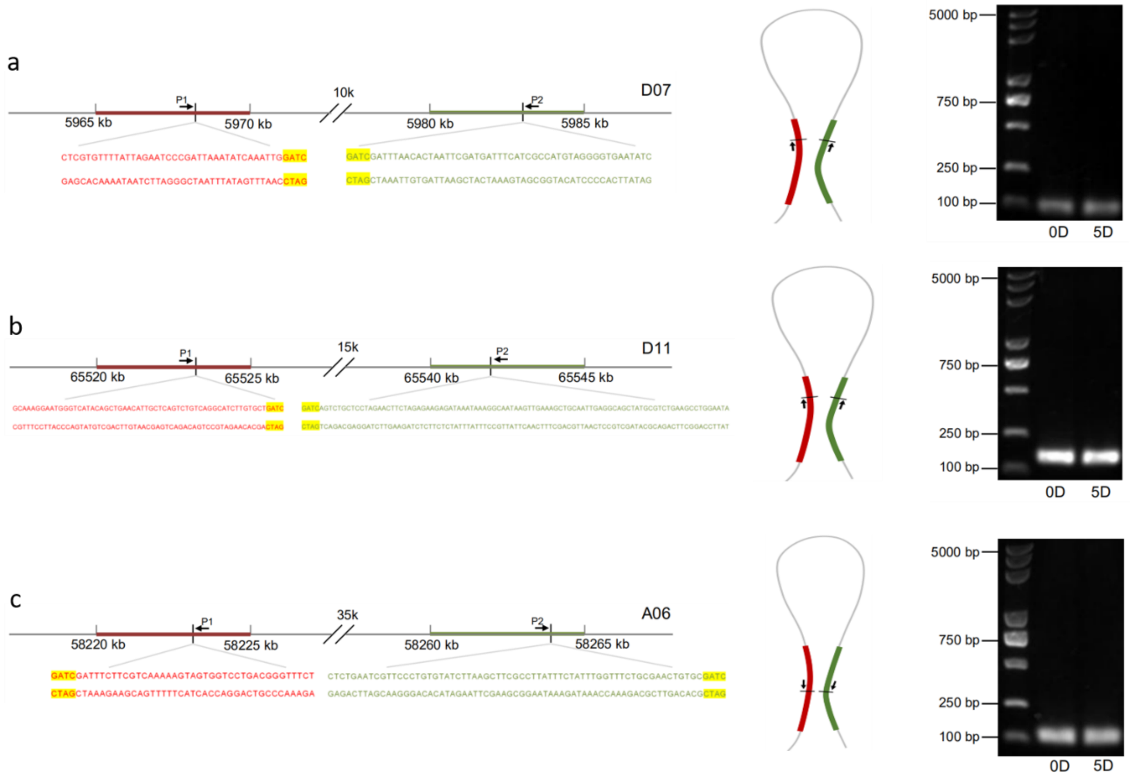

Fig. S21

PCR validation of three chromatin loops.

The DpnII digestion and ligation of chromatin associated with three loops on Chr D07 (a), D11 (b) and A06 (c) were validated using PCR experiment, with samples of 0 DPA and 5 DPA. The PCR primer sites were indicated by P1 and P2 for each loop.

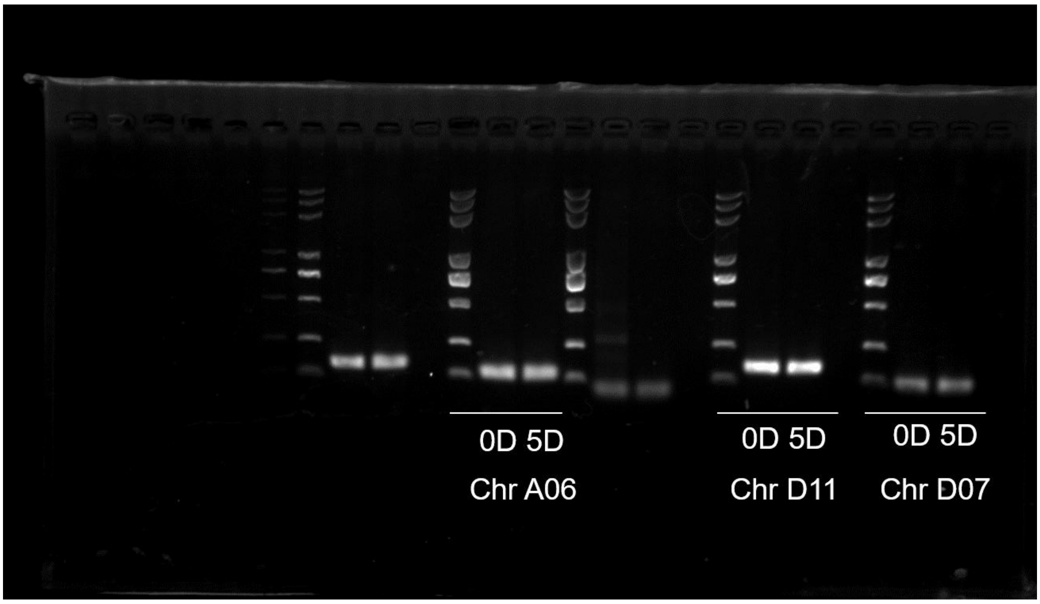

Fig. S22

The uncropped image of PCR validation of three chromatin loops shown in Fig. S21.

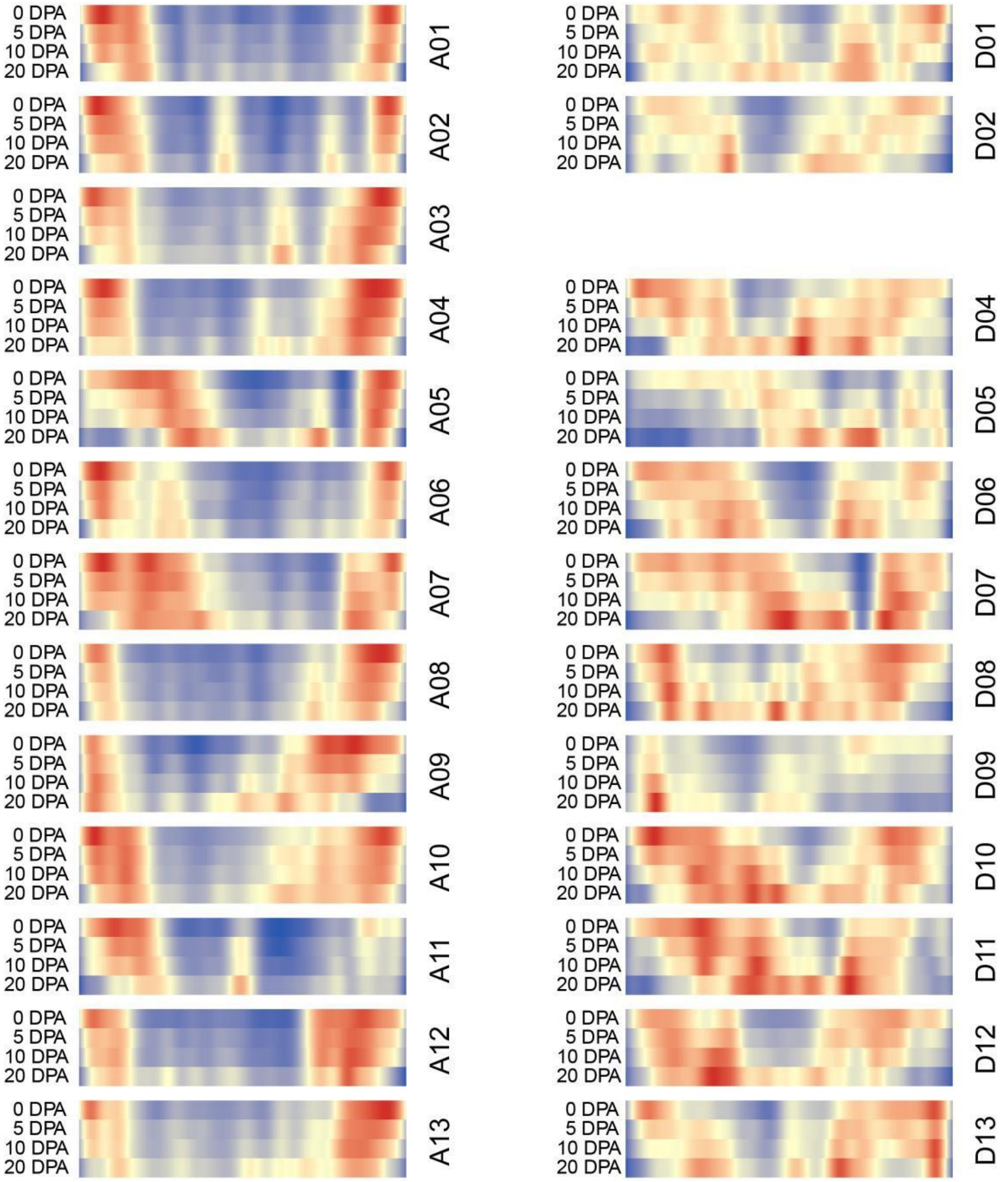

Fig. S23

The loop anchor density in each chromosome during fiber development. More interactions are shown in red and less interactions are shown in blue. The data of chromosome D03 are shown in **Fig. 7**.

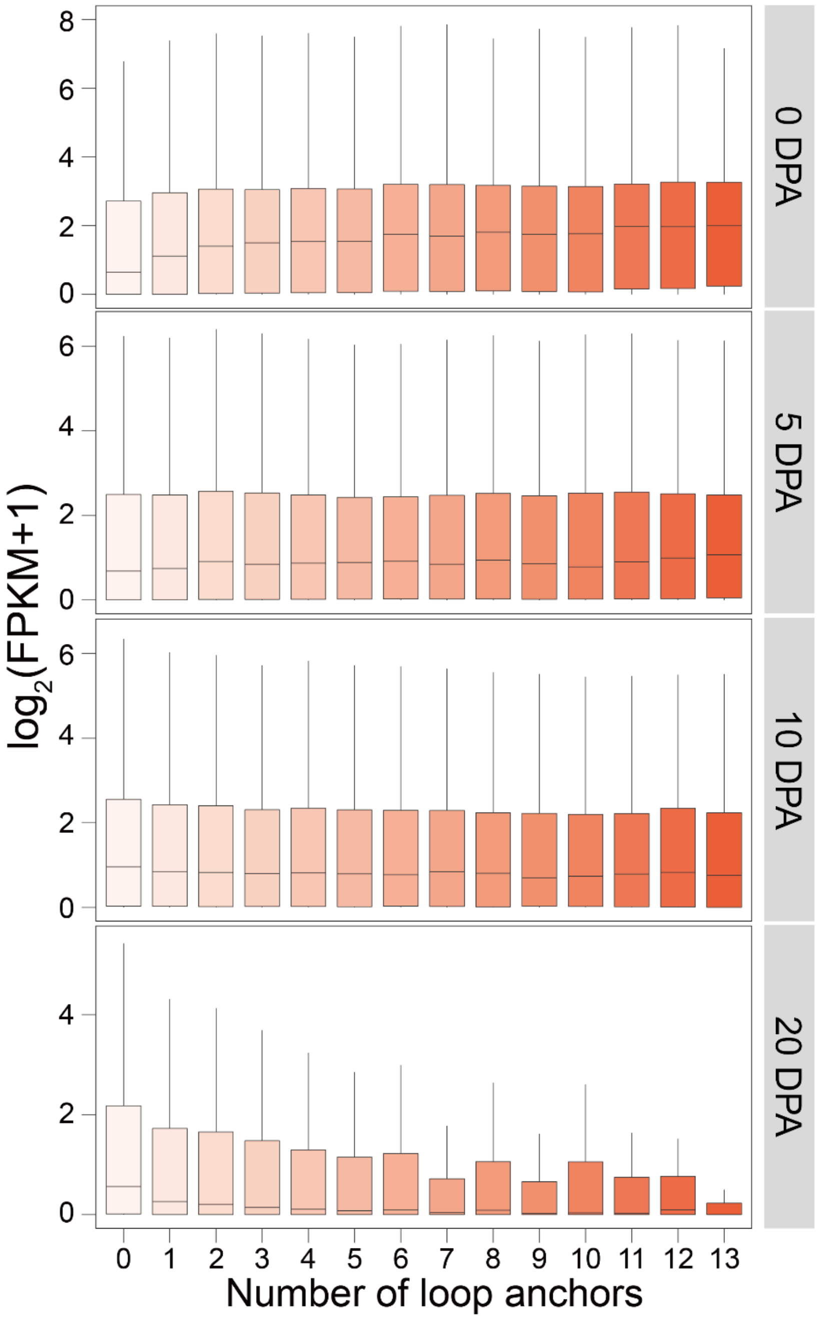

Fig. S24

Box plot showing the relationship between the expression level of genes and the number of loop anchors.

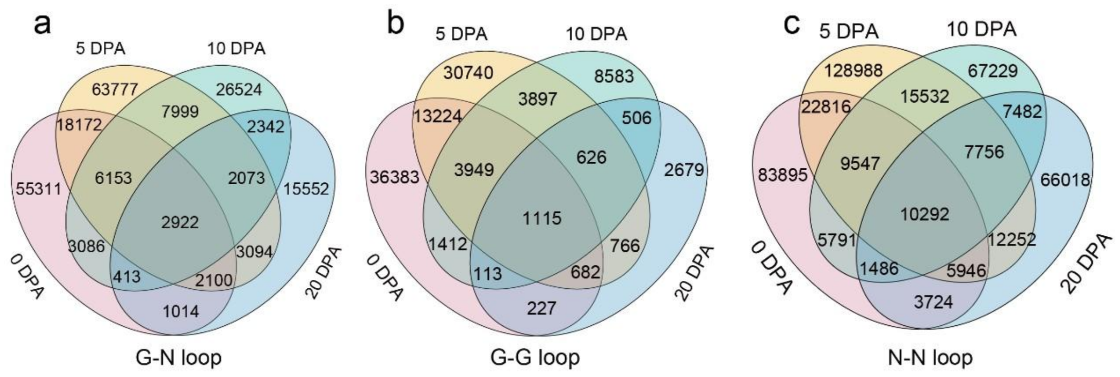

Fig. S25

The number of the three types of loops at four stages.

a, The Venn plot shows the dynamic and conserved number of G-G loops at four stages. **b,** The Venn plot shows the dynamic and conserved number of G-N loops at four stages. **c,** The Venn plot shows the dynamic and conserved number of N-N loops at four stages.

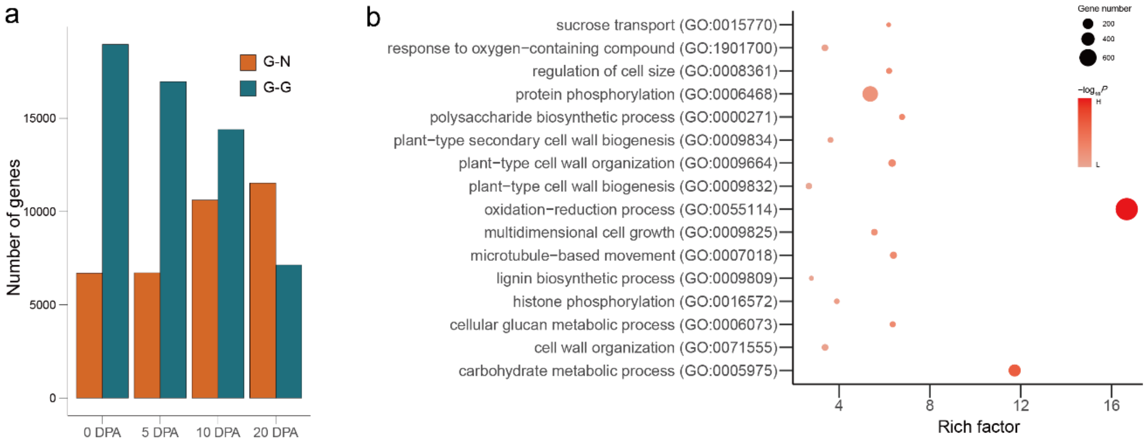

Fig. S26

The number and enriched GO terms of genes that were located in loop anchors and possibly associated with fiber development.

a, The bar plot shows the number of genes that can be contained in two types of loops.

b, The point plot shows the enriched GO terms of genes that were located in dynamic loop anchors between adjacent periods.

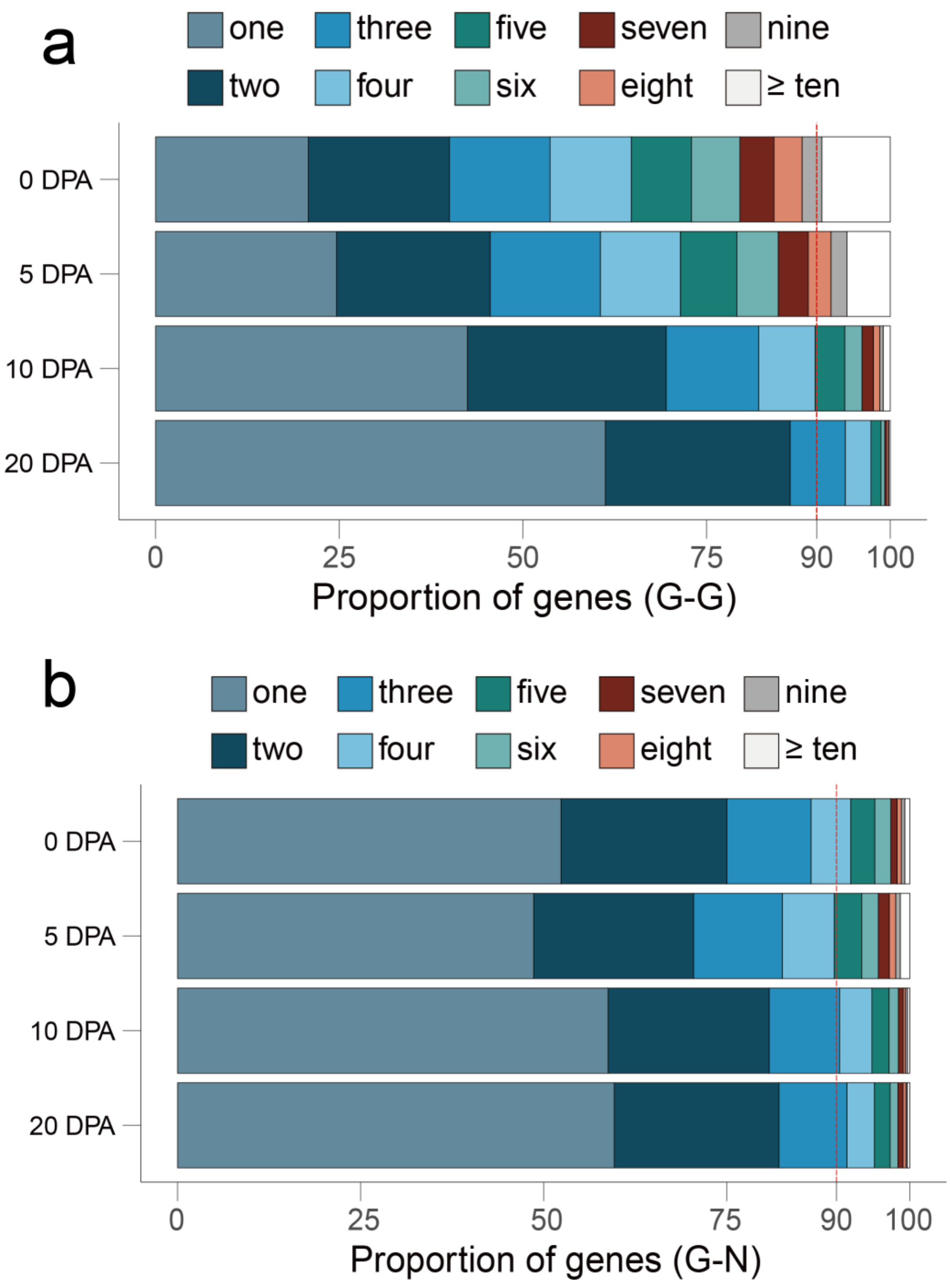

Fig. S27

Analysis of genes linked by different types of loops.

a, The bar shows the proportion of genes that can be linked by different numbers of G-G loops. **b,** The bar shows the proportion of genes that can be linked by different numbers of G-N loops.

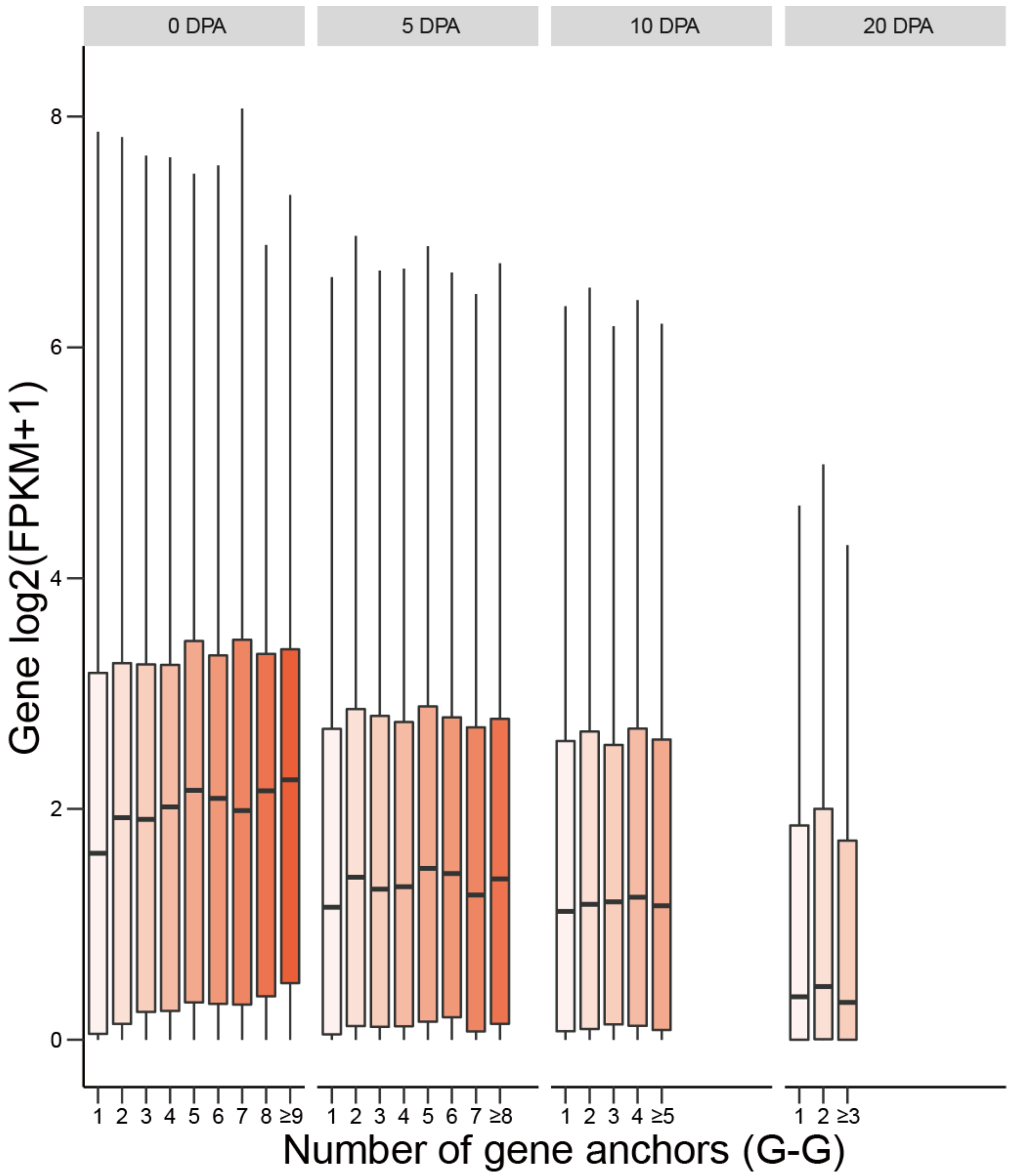

Fig. S28

The relationship between the expression level of genes and the number of associated G-G loops.

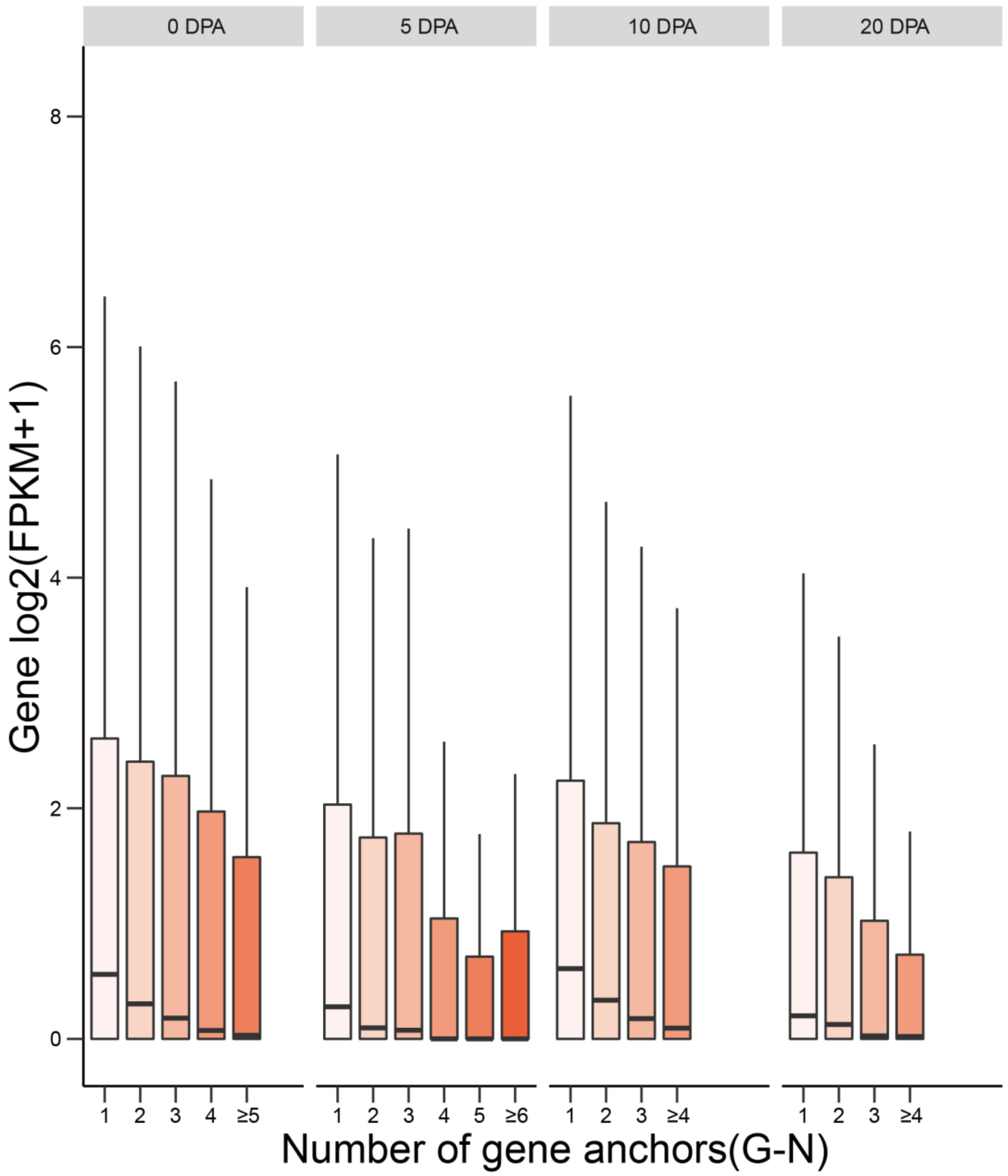

Fig. S29

The relationship between the expression level of genes and the number of associated G-N loops.

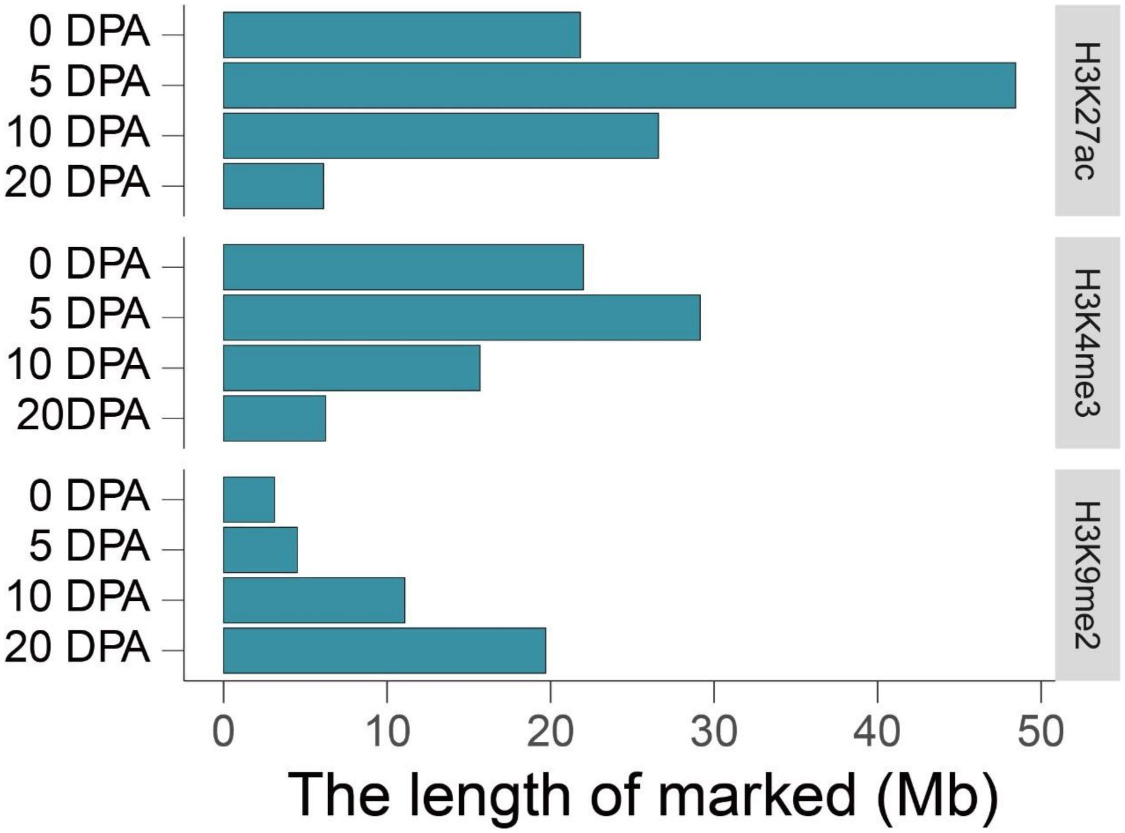

Figure. S30

The length of regions with different histone modifications.

The regions refers to the non-gene regions connected to the G-N loops.

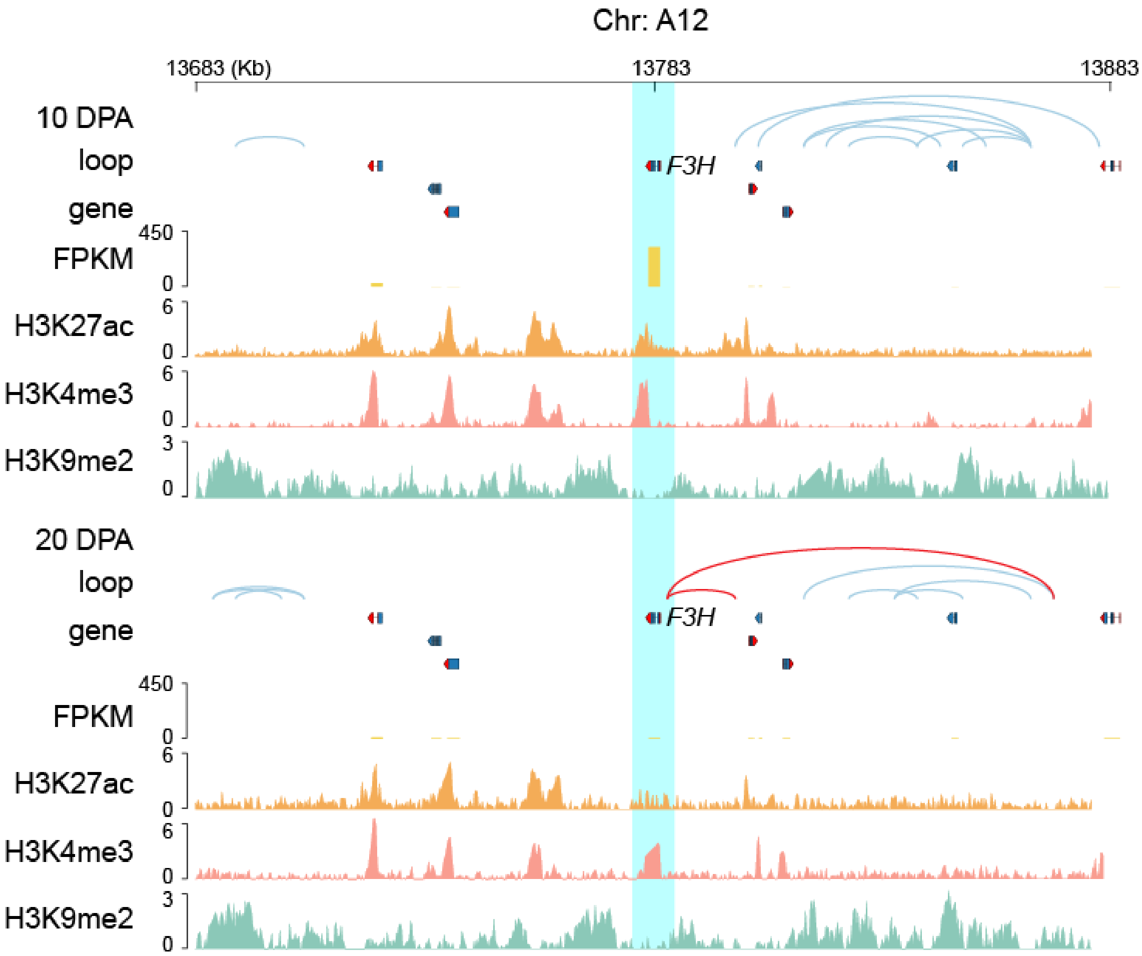

Fig. S31

Chromatin contacts, expression levels and epigenetic states around 100 Kb of *F3H* gene at 10 DPA and 20 DPA. The red lines represent loops related to *F3H*. The *F3H* interacts with the region that contained H3K4me3 modification while its expression level was reduced from 10 DPA to 20 DPA.

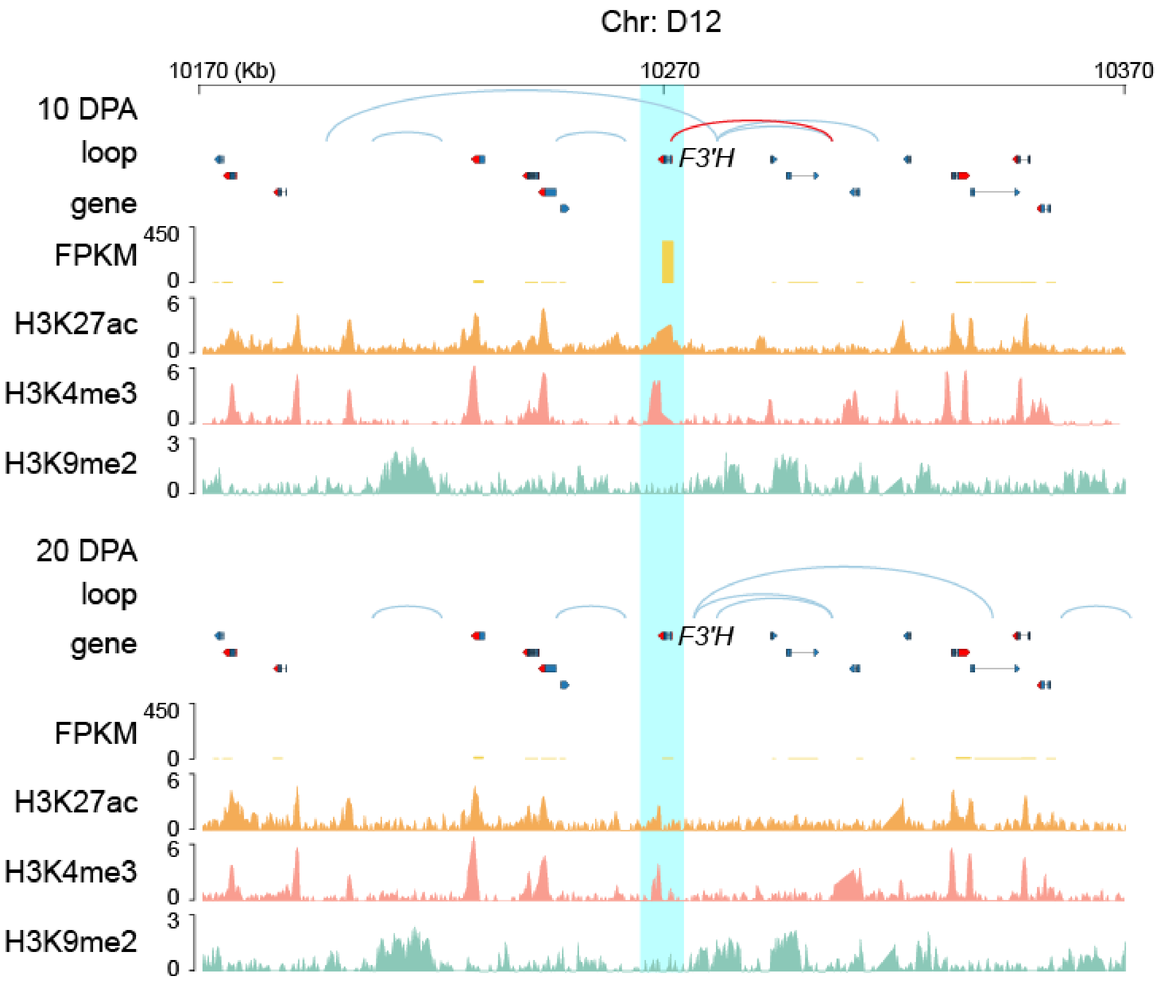

Fig. S32

Chromatin contacts, expression levels and epigenetic states around 100 Kb of *F3'H* gene at 10 DPA and 20 DPA. The red lines represent loops related to *F3'H*. The interaction of *F3H* with the regions that contained H3K4me3 modification was lost while its expression level was reduced from 10 DPA to 20 DPA.

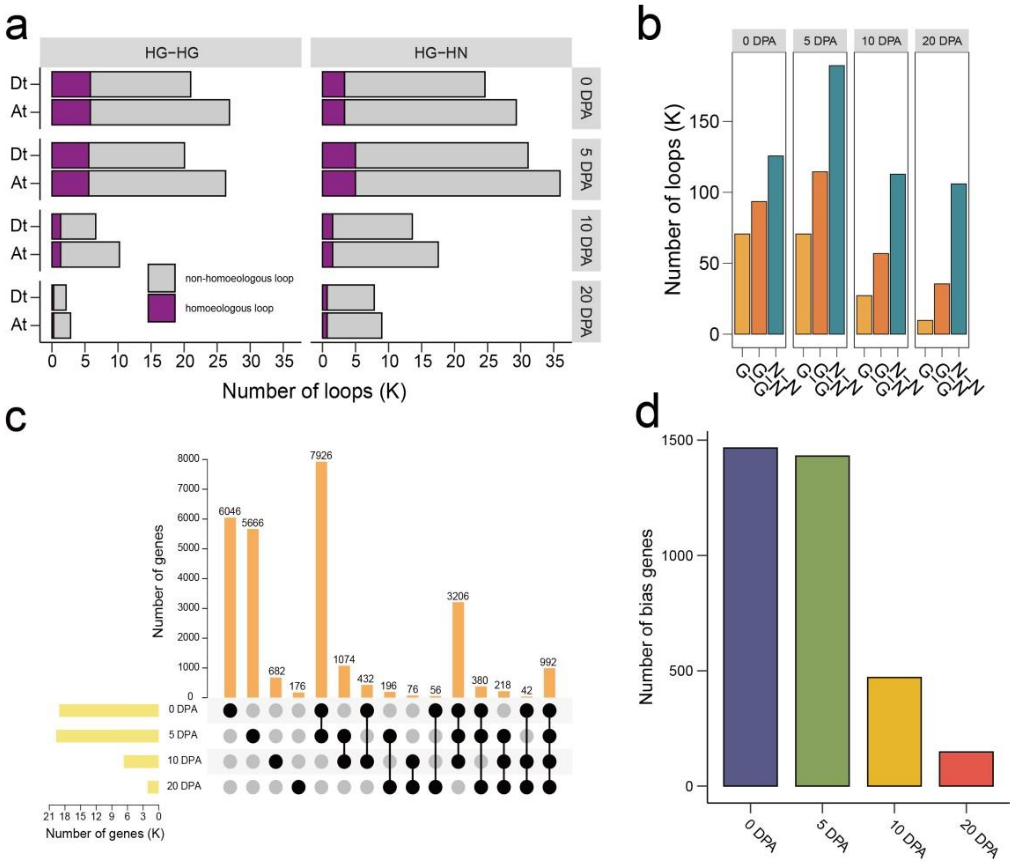

Fig. S33

Analysis of homoeologous loops associated genes.

a, The number of three different types of loops during fiber development. **b,** The distribution of the genes contained in homoeologous loops during fiber development. purple refers to homoeologous loops, grey represents non-homoeologous loops. **c,** The number of genes with expression bias that can be linked by homoeologous loops. **d,** Bar plot showing the number of loops that are connected to homoeologous genes.

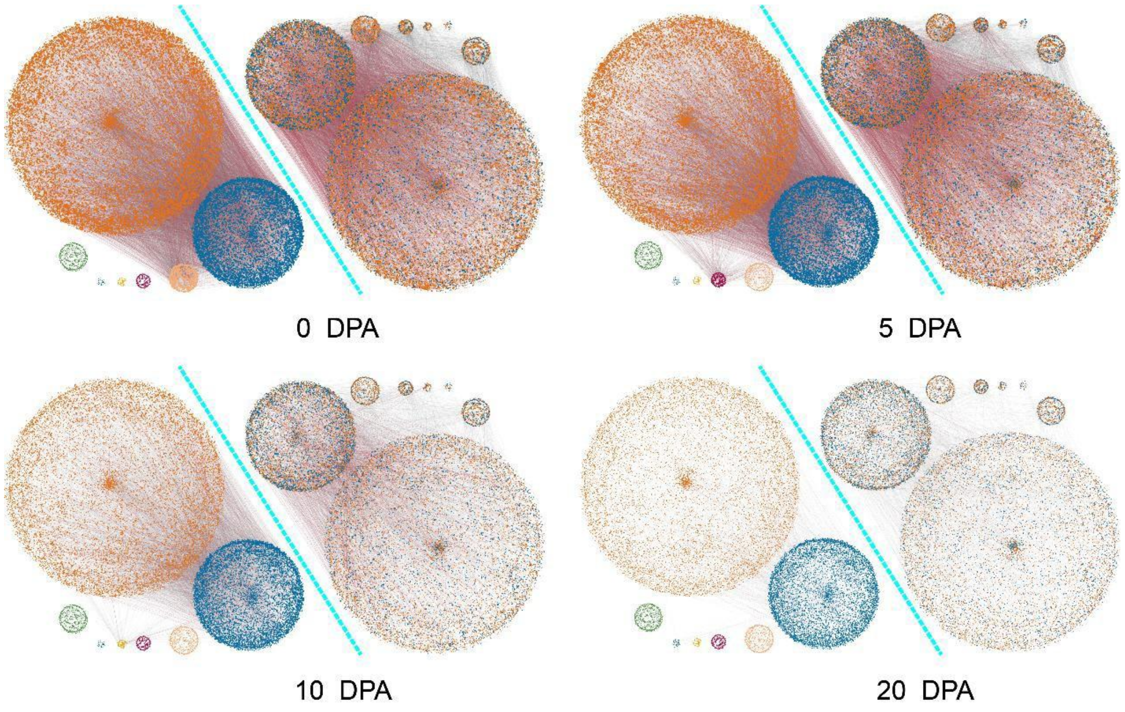

Fig. S34

Chromatin loops-mediated interaction networks of homoeologous genes. The red lines refer to homoeologous loops. The grey lines refer to non-homoeologous loops.

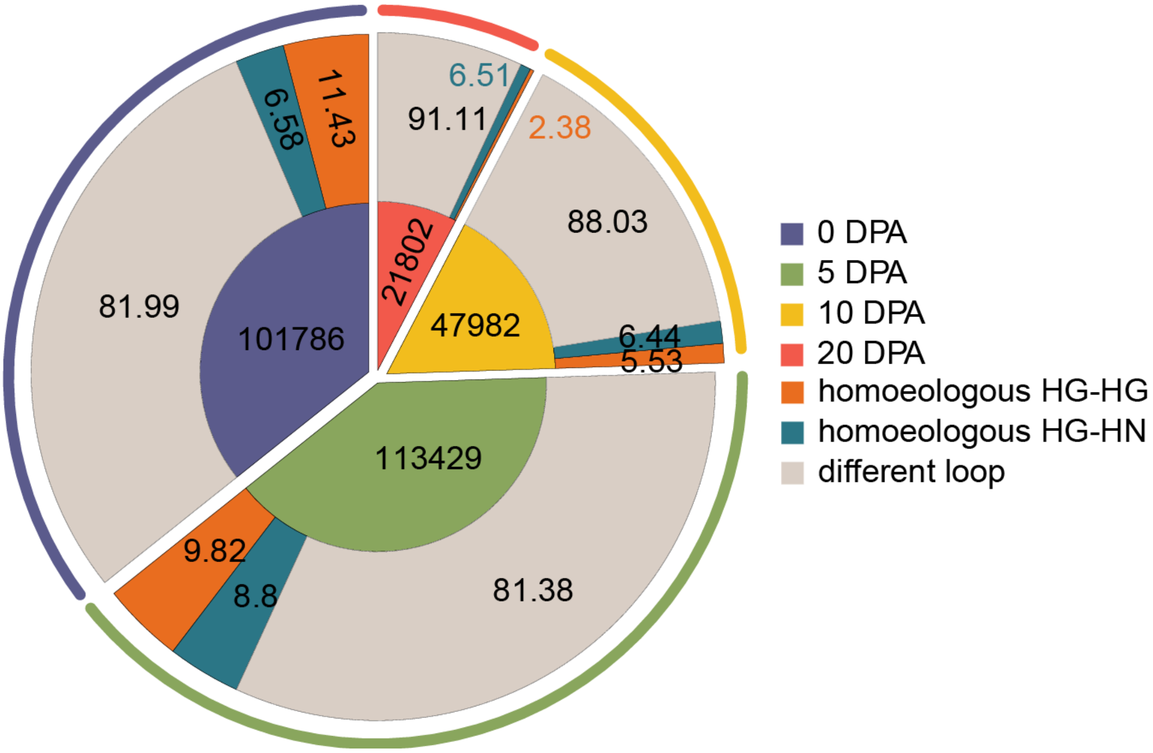

Fig. S35

The pie chart shows the number of HG-HG and HG-HN loops and the proportion of homoeologous HG-HG loops and homoeologous HG-HN loops. The inner circle indicates the number of HG-HG loops and HG-HN loops at four stages. The middle ring indicates the proportion of three different types of loops at each stage. The outermost ring shows the stages.
