# Supplementary material for: Dynamic 3D genome architecture of cotton fiber reveals subgenome-coordinated chromatin topology for 4-staged single-cell differentiation
Source: Genome Biol. 2022 Feb 3;23:45. doi: 10.1186/s13059-022-02616-y (PMC8812185; doi:10.1186/s13059-022-02616-y)
Supplement: Supplementary file 15 — Additional file 15. Review history. [file 13059_2022_2616_MOESM15_ESM.docx]

Review History

**First round of review**

**Reviewer 1**

**Are you able to assess all statistics in the manuscript, including the appropriateness of statistical tests used?**

Yes, and I have assessed the statistics in my report.

**Comments to author:**

One of the most important traits in the cotton crop is fiber. Even though extensive studies on cotton genomes and transcriptome, the 3D genome architecture, in particular to 3D structures related to fiber development, is far from well-documented. This manuscript by Pei et., al presents the first 3D genome study at different developmental stages of fiber in cotton, showing dynamic changes of chromatin topology during fiber development and significant difference between the two subgenomes at multiple levels, including chromatin loops and transcriptional regulation. In addition, the authors proposed that differential TE activity contributed to dynamic changes of 3D structure between homoeologous chromosomes, leading to expression bias. Overall, the manuscript is well-written and I read this with great interest. Below are my comments:

1. Fig.3a shows dynamic switching of A/B compartment during four developmental stages. However, it is difficult to detect the switching regions visually. Obviouse switching regions should be marked in the figure.

2. Lines 262-263, the authors identified many differentially expressed genes with TAD reorganization. Do they have important functions related to fiber development? A brief summary is needed here.

3. Fig. 5h shows the biased expression between genes located in TAD boundary and randomly distributed genes. However, little information was provided regarding the methods used here. How were these genes randomly selected? And how many genes were included in this analysis?

4. Fig. 5g shows a case study of biased expression between two subgenomes, which is likely linked to TE activity and altered 3D structure. The data presented here convinced me; however, functional implications of this (or these) genes should be mentioned here.

5. What is the coverage of Hi-C sequencing data? I did not see the details in METHODS.

6. How do the authors compare Hi-C and ChiA-PET technologies? I generally think that the ChiA-PET is more powerful with less noisy signals to investigate the chromatin contact.

**Reviewer 2**

**Are you able to assess all statistics in the manuscript, including the appropriateness of statistical tests used?**

Yes, and I have assessed the statistics in my report.

**Comments to author:**

The manuscript by Pei et al. reports 3D genome topology of cotton fiber for 4-staged single-cell differentiation by combing in-situ HiC, ChIP-seq and RNA-seq. The authors studied the changes of gene expression, chromosome compartments, TADs and loops during cotton fiber development and also elucidated the divergences between At and Dt subgenomes. There's no doubt that this is a work of important scientific significance. However, in my opinion, the data analysis needs to be improved, which will likely change some conclusions.

Major points:

1. More analysis should be included to qualify the data. eg. for the HiC data, the statistics of alignment reads, percentage of cis-short, cis-long and trans contact pairs; for the RNA-seq data, a PCA analysis will better describe the differences among samples; f the ChIP-seq data, the FRiPs of each sample, the distribution of reads around genes (H3K27ac and H3K4me3) or across whole chromosomes (for H3K9me2).

2. The authors should prove the quality of those HiC data from different tissues were comparable before performing the comparation among different samples. The TADs, loops and compartments analysis are affected by the quality of HiC assay (Yardimci, 2019, https://doi.org/10.1186/s13059-019-1658-7). For example, the differential expressed genes (LINE 137) from 5vs10 DPA are among the lowest, while in the AtoB/BtoA compartment analysis, 5vs10 DPA showed the most alterations (Fig 6). The conflictions are biological or technical need to be stated. I would suggest the authors to try some packages like TADcompare, HiC-DC+, diffloop etc, which are designed to perform differential analysis.

Other points,

1. Fig 1a and 1b, the counts of expressed genes are decreased along with developmental progresses, while the counts of the activating marker H3K27ac peaks are much less in 0 than that in 5DPA and 10DPA. The count differentiations of ChIP-seq peaks are more like a technical problem.

2. Fig 2b,2c,2d, the distance distributions of contact pairs were usually affected by the quality of HiC data and considered as an index of data quality, it can not be used to make conclusions about whether there're more long distance or trans interactions.

3. Fig5g,7h,7j,8d, for the examples showing the genomic coverage images, an "input" sample should be included.

4. For sentences with a series of number, it would be better to change another way to present. Eg. LINE 137, "we identified 10,155, 2,162, 7,188 differentially expressed genes in three comparisons (0 vs 5 DPA, 138 5 vs 10 DPA, 10 vs 20 DPA)" to "we identified 10,155 (0 vs 5DPA), 2,162 (5 vs 10 DPA), 7,188 (10 vs 20 DPA) differentially expressed genes in three comparisons" would help the reader to understand.

5. In LINE 668, "only those with expression fold change larger than one and FDR less than 0.01 were retained", "larger than one" should be "log2(fold_change) > 1"?

**Reviewer 3**

**Are you able to assess all statistics in the manuscript, including the appropriateness of statistical tests used?**

Yes, and I have assessed the statistics in my report.

**Comments to author:**

In this manuscript, Pei et al., established the dynamic three-dimensional (3D) genome architecture of the allotetraploid cotton fiber. Authors showed that a subgenome-relayed switching of the chromatin compartment from active to inactive and that this was coupled with the silencing of developmentally repressed genes, pinpointing subgenome-coordinated contribution to fiber development. Authors identified 10,571 topologically associating domains (TADs), of which 25.6% were specifically organized in different stages and 75.23% were subject to partition or fusion. Interestingly authors observed that , dissolution of intricate TAD cliques showing long-range TAD-TAD interactions represented a prominent characteristic at the later developmental stage. Authors also observed that dynamic chromatin loops mediates the rewiring of gene regulatory networks that exhibited a significant difference between the two subgenomes, implicating expression bias of homoeologous genes. Overall, the topic is very interesting and data quality is very good. While I liked the data presented and believe this is significant progress in the field, there are a number of points that should be addressed:

1. Of all the chromatin interactions described, TADs are the best characterized, as they are visually recognizable as high interaction squares along the diagonal in Hi-C matrices, with a relatively low resolution (Maass et al., 2019). They are defined as 3D structures where the chromatin regions included within a TAD interact with each other in cis with a higher frequency than with chromatin outside it. In addition to this, animal TADs which were the first described are isolated units where genes inside the same TAD are co-regulated (Dixon et al., 2012, 2016). In plants, it was shown that genes are enriched in TAD borders whereas TE are enriched inside TAD-like structures ((Liu et al., 2017; Dong et al., 2018 ; Wang et al., 2018 ; Concia et al., 2020 ; in this article also see figure 5 for example). This suggests that TADs in animals and in plants are different in nature and function. In this context I suggest authors to replace TAD by TAD like structure in the text.

2. For the figure 2B, C and D authors should separate gene rich and gene poor region, which basically will segregate pericentromeric region and arms. This will allow the authors to see if the difference is mainly due to a reorganization of the eu- or hetero-chromatin or both.

3. Figure 3 It would be interesting if the authors can analyze the dynamics of the H3K27ac, H3K9me2 and H3K4me3 in the compartment that switch during fiber development.

4. Figure 5 A,B and G: the HiC map is saturated and does not allow us analyze anything. Authors should correct it and add a line with insulation values.

5. The part of the work related to the analysis of the relationship between loop dynamics and gene expression is weak and only correlative. For that reason authors should tone down their conclusions which are not supported by functional analysis. For example authors wrote that "These data together reveal the dynamic rewiring of chromatin loops and implicate their regulatory role in gene expression". Correlation does not mean cause / consequence effect.

6. Another weak point is the fact that authors never confirmed any of their observation using either a cytological approach like FISH or at least by doing 3C-QPCR.

7. The paper contains a large amount of data and numerous techniques. As such, it is not easy to read. Results would benefit if each section would start with a hypothesis or explanation of what is expected and what that specific analysis means rather than relying on the reader to go to M&M or publications to get the background.

**Authors Response**

**Point-by-point responses to the reviewers’ comments:**

**Reviewer #1:**

One of the most important traits in the cotton crop is fiber. Even though extensive studies on cotton genomes and transcriptome, the 3D genome architecture, in particular to 3D structures related to fiber development, is far from well-documented. This manuscript by Pei et., al presents the first 3D genome study at different developmental stages of fiber in cotton, showing dynamic changes of chromatin topology during fiber development and significant difference between the two subgenomes at multiple levels, including chromatin loops and transcriptional regulation. In addition, the authors proposed that differential TE activity contributed to dynamic changes of 3D structure between homoeologous chromosomes, leading to expression bias. Overall, the manuscript is well-written and I read this with great interest. Below are my comments:

1. Fig.3a shows dynamic switching of A/B compartment during four developmental stages. However, it is difficult to detect the switching regions visually. Obviouse switching regions should be marked in the figure.

*Response: Thanks very much for your suggestion. We marked the dynamic switching regions of A/B compartment in Figure 3a, and the data were also shown in Figure S8- 10.*

2. Lines 262-263, the authors identified many differentially expressed genes with TAD reorganization. Do they have important functions related to fiber development? A brief summary is needed here.

*Response: Thanks very much for the insightful comments. We counted the differentially expressed genes around dynamic TAD boundaries and performed the GO enrichment analysis on these genes, which showed that they were enriched in a few biological processes such as carbohydrate metabolic process and sucrose metabolic process (shown below). More details can be found in Figure S16.*

3. Fig. 5h shows the biased expression between genes located in TAD boundary and randomly distributed genes. However, little information was provided regarding the methods used here. How were these genes randomly selected? And how many genes were included in this analysis?

*Response: Thanks very much for your comments. We first counted the number of homoeologous gene pairs that were located in boundary or interior (3904, 3828, 3659, 3808 in four periods) and then randomly selected the corresponding number of homoeologous gene pairs in each period. After that, we counted the number of homoeologous gene pairs with biased expression in these two groups separately (1504/3904, 1154/3828, 1031/3659, 1449/3808; 351/3904, 231/3828, 175/3659, 392/3808).*

4. Fig. 5g shows a case study of biased expression between two subgenomes, which is likely linked to TE activity and altered 3D structure. The data presented here convinced me; however, functional implications of this (or these) genes should be mentioned here.

*Response: Thanks very much for your comments. We first counted the number of homoeologous gene pairs that were located in boundary or interior (3904, 3828, 3659, 3808 in four periods) and then randomly selected the corresponding number of homoeologous gene pairs in each period. After that, we counted the number of homoeologous gene pairs with biased expression in these two groups separately (1504/3904, 1154/3828, 1031/3659, 1449/3808; 351/3904, 231/3828, 175/3659, 392/3808.*

5. What is the coverage of Hi-C sequencing data? I did not see the details in METHODS.

*Response: Thanks for your suggestion. We provided the coverage of Hi-C sequencing data in Methods.*

6. How do the authors compare Hi-C and ChiA-PET technologies? I generally think that the ChiA-PET is more powerful with less noisy signals to investigate the chromatin contact.

*Response: Thanks very much for your suggestion. We agree with you that ChIA-PET is indeed more powerful for analyzing the bona fide regulatory relationships at the level of chromatin loops, but this method is powerful in identifying loops associated with a specific protein. Theoretically, Hi-C can be used to identify all chromatin loops. Here, we want to investigate multi-hierarchical reorganization of chromatin architecture during fiber development, so we determined to use the Hi-C method.*

**Reviewer #2:**

The manuscript by Pei et al. reports 3D genome topology of cotton fiber for 4-staged single-cell differentiation by combing in-situ HiC, ChIP-seq and RNA-seq. The authors studied the changes of gene expression, chromosome compartments, TADs and loops during cotton fiber development and also elucidated the divergences between At and Dt subgenomes. There's no doubt that this is a work of important scientific significance. However, in my opinion, the data analysis needs to be improved, which will likely change some conclusions.

Major points:

1. More analysis should be included to qualify the data. eg. for the HiC data, the statistics of alignment reads, percentage of cis-short, cis-long and trans contact pairs; for the RNA-seq data, a PCA analysis will better describe the differences among samples; f the ChIP-seq data, the FRiPs of each sample, the distribution of reads around genes (H3K27ac and H3K4me3) or across whole chromosomes (for H3K9me2).

*Response: Thanks very much for your suggestions. More details on the statistical information of Hi-C data can be found in Table S5. For RNA-Seq data, we performed PCA analysis using the normalized number of reads on genes and the results showed a very good clustering between biological replicates and low similarity between samples from different periods. For ChIP-Seq data, we calculed the FRiPs of each sample and analyzed the distribution of reads (H3K27ac and H3K4me3) around genes and ChIP peaks (H3K27ac, H3K4me3 and H3K9me2) at the genome-wide level, respectively. These results are shown below and Figure S1.*

2. The authors should prove the quality of those HiC data from different tissues were comparable before performing the comparation among different samples. The TADs, loops and compartments analysis are affected by the quality of HiC assay (Yardimci, 2019, https://doi.org/10.1186/s13059-019-1658-7). For example, the differential expressed genes (LINE 137) from 5vs10 DPA are among the lowest, while in the AtoB/BtoA compartment analysis, 5vs10 DPA showed the most alterations (Fig 6). The conflictions are biological or technical need to be stated. I would suggest the authors to try some packages like TADcompare, HiC-DC+, diffloop etc, which are designed to perform differential analysis.

*Response: Thanks very much for your valuable suggestions on this manuscript. We have used specialized tools to compare the 3D genome structures. TADcompare was used to compare the TADs between adjacent periods. The result is very consistent with our previous analysis (76.98% vs 74.4%). More details can be found in Table S13. Actually, our method was also described in a few previous studies (Cell 2021, 184:723-740; Nat Commun 2021, 12:1-17; Cell Rep 2019, 29:4200-4211). Detailed information of Hi-C data for each sample is presented in Table S5 and valid interaction pairs of samples are almost equal, which ensures that our comparison results are not affected by sequencing depth.*

*In terms of the RNA-Seq data and A/B compartment between 5 DPA and 10 DPA, we should first recognize that the developmental status of fibers at 5 DPA and 10 DPA are relatively close. The shift in the A/B compartment is a change shown in a very large hierarchical structure, while the change in gene expression level is accompanied by very fine regulatory changes. In fact, the compartment switching has only a very slight correlation with gene expression, which is observed in both animals and plants. Considering this, we did not directly say compartment swithing contributed to differential expression of genes throughout the manuscript.*

Other points,

1. Fig 1a and 1b, the counts of expressed genes are decreased along with developmental progresses, while the counts of the activating marker H3K27ac peaks are much less in 0 than that in 5DPA and 10DPA. The count differentiations of ChIP-seq peaks are more like a technical problem.

*Respones: Thanks very much for your comments. As you know, cotton ovules (0 DPA) are composed of different types of cells, while cotton fibers are pure singlecells (5 DPA, 10 DPA and 20 DPA). Cotton fiber cells are differentiated from epidermis of cotton ovules around 0 DPA, so in this study we only used cotton ovules at 0 DPA as a control to compare with elongating fibers at 5, 10 and 20 DPA. If comparing the data between cotton ovules (0 DPA) and fibers (5 DPA, 10 DPA and 20 DPA), at the current stage we indeed cannot explain why the different patterns between the number of expressed genes and the number of H3K27ac peaks appeared. We deduce that the tissue difference might be responsible for this without solid evidence. In this manuscript, we mainly illustrated the relationship between dynamic chromatin architecture and gene expression regulation during fiber elongation (5 DPA, 10 DPA and 20 DPA).*

2. Fig 2b,2c,2d, the distance distributions of contact pairs were usually affected by the quality of HiC data and considered as an index of data quality, it can not be used to make conclusions about whether there're more long distance or trans interactions.

*Response: Thanks very much for your suggestion. As you mentioned that whether there are more long distance or trans interactions need more experimental data to prove it, we revised these discriptions as suggested in the manuscript.*

3. Fig5g,7h,7j,8d, for the examples showing the genomic coverage images, an "input" sample should be included.

*Respones: Thanks very much for your suggestion. Indeed, we have already corrected the ChIP-Seq data with input, so we did not show this in these figures. We have described the details for how we processed the ChIP-Seq data in the Method.*

4. For sentences with a series of number, it would be better to change another way to present. Eg. LINE 137, "we identified 10,155, 2,162, 7,188 differentially expressed genes in three comparisons (0 vs 5 DPA, 138 5 vs 10 DPA, 10 vs 20 DPA)" to "we identified 10,155 (0 vs 5DPA), 2,162 (5 vs 10 DPA), 7,188 (10 vs 20 DPA) differentially expressed genes in three comparisons" would help the reader to understand.

*Respones: Thanks for your helpful suggestion. We have revised these sentences as suggested throughout the manuscript.*

5. In LINE 668, "only those with expression fold change larger than one and FDR less than 0.01 were retained", "larger than one" should be "log2(fold_change) > 1"?

*Response: Thanks for your suggestion. We have revised it as suggested.*

**Reviewer #3:**

In this manuscript, Pei et al., established the dynamic three-dimensional (3D) genome architecture of the allotetraploid cotton fiber. Authors showed that a subgenome-relayed switching of the chromatin compartment from active to inactive and that this was coupled with the silencing of developmentally repressed genes, pinpointing subgenome-coordinated contribution to fiber development. Authors identified 10,571 topologically associating domains (TADs), of which 25.6% were specifically organized in different stages and 75.23% were subject to partition or fusion. Interestingly authors observed that , dissolution of intricate TAD cliques showing long-range TAD-TAD interactions represented a prominent characteristic at the later developmental stage. Authors also observed that dynamic chromatin loops mediates the rewiring of gene regulatory networks that exhibited a significant difference between the two subgenomes, implicating expression bias of homoeologous genes. Overall, the topic is very interesting and data quality is very good. While I liked the data presented and believe this is significant progress in the field, there are a number of points that should be addressed:

1. Of all the chromatin interactions described, TADs are the best characterized, as they are visually recognizable as high interaction squares along the diagonal in Hi-C matrices, with a relatively low resolution (Maass et al., 2019). They are defined as 3D structures where the chromatin regions included within a TAD interact with each other in cis with a higher frequency than with chromatin outside it. In addition to this, animal TADs which were the first described are isolated units where genes inside the same TAD are co-regulated (Dixon et al., 2012, 2016). In plants, it was shown that genes are enriched in TAD borders whereas TE are enriched inside TAD-like structures ((Liu et al., 2017; Dong et al., 2018 ; Wang et al., 2018 ; Concia et al., 2020 ; in this article also see figure 5 for example). This suggests that TADs in animals and in plants are different in nature and function. In this context I suggest authors to replace TAD by TAD like structure in the text.

*Response: Thanks very much for your suggestion and detailed explanations. We have replaced TAD by TAD-like structure in this manuscript, in order to show that TADs in animals and plants are different in nature and function.*

2. For the figure 2B, C and D authors should separate gene rich and gene poor region, which basically will segregate pericentromeric region and arms. This will allow the authors to see if the difference is mainly due to a reorganization of the eu- or hetero-chromatin or both.

*Response: Thanks very much for your suggestion. We have performed the analysis by separating gene-rich and gene-poor regions as suggested, and we found that the result (below) showed no significant difference from our previous analysis. It is noteworthy that the difference of compactness between At and Dt is pronounced in gene-poor regions. Please find more details in Figure S6.*

3. Figure 3 It would be interesting if the authors can analyze the dynamics of the H3K27ac, H3K9me2 and H3K4me3 in the compartment that switch during fiber development.

*Response: Thanks very much for your suggestion. The points you raise really deserve further study, and we want to clarify the situation in this study. First of all, the A/B compartment switching regions occupied a very small proportion of chromosomes (0.43%~2.69%) that covered a limited number of dynamic ChIP peaks. Second, it is necessary to consider the effect of the properties of the dynamic ChIP peaks and the compartment itself on the results. The active modifications (H3K27ac, H3K4me3) become progressively less during fiber elongation, while the inactive modification (H3K9me2) increases. Finally, the switching of the A/B compartment is observed at a very sparse resolution of chromatin status, and ChIP peak changes occur at very small regions. We performed analysis based on your suggestion, but we did not observe very strong correlation between compartment status switching and changes of ChIP peaks, possibly due to the small number that are overlapped between them.*

4. Figure 5 A,B and G: the HiC map is saturated and does not allow us analyze anything. Authors should correct it and add a line with insulation values.

*Response: Thanks very much for your suggestion. We have corrected the Hi-C map and added a line with insulation values in Figures 4E, 5A, B, G and S15.*

5. The part of the work related to the analysis of the relationship between loop dynamics and gene expression is weak and only correlative. For that reason authors should tone down their conclusions which are not supported by functional analysis. For example authors wrote that "These data together reveal the dynamic rewiring of chromatin loops and implicate their regulatory role in gene expression". Correlation does not mean cause / consequence effect.

*Response: Thanks very much for your suggestion. We performed GO enrichment analysis of differentially expressed genes associated with dynamic loops. The GO terms associated with fiber development were shown below (please see Figure S25b). At the same time, we toned down the conclusion that dynamic loops correlated with gene expression in this manuscript. This sentence was changed to “These data together reveal the dynamic rewiring of chromatin loops and inform future study to explore their putative regulatory role in gene expression.”*

6. Another weak point is the fact that authors never confirmed any of their observation using either a cytological approach like FISH or at least by doing 3C-QPCR.

*Response: Thanks very much for your suggestion. As you know, it is very challenging to perform the FISH experiment in cotton fiber which is very different from other tissues such as leaf. As far as we know, there are no very good FISH results for cotton fiber in the cotton community. In this study, we aimed at uncovering the general principle of dynamic organization of chromatin structure during staged fiber development, so we did not perform case-by-case molecular validation. In the revised manuscript, we validated three loops using PCR experiment (Please see Figure S21). In the future, we will perform more analyses showing how 3D genome structure is associated with a specific trait such as fiber length. For example, more functional validation will be performed for TAD-like structure boundary or loop-involved genes in GWAS QTL using genome-edited plants, but it should take a long time to fulfill such studies. We sincerely hope that you could understand this point.*

7. The paper contains a large amount of data and numerous techniques. As such, it is not easy to read. Results would benefit if each section would start with a hypothesis or explanation of what is expected and what that specific analysis means rather than relying on the reader to go to M&M or publications to get the background.

*Response: Thanks very much for your suggestion. To help readers better understand the results in this manuscript, we have provided more descriptions for a hypothesis or explanation of what is expected and what a specific analysis means.*

**Second round of review**

**Reviewer 1**

The authors have addressed all of my concerns and I have no further comments.

**Reviewer 2**

Thanks for the authors' responses. They have answered all my concerns.

**Reviewer 3**

The authors fully addressed all concerns.
